# Metabolome and Whole-Transcriptome Analyses Reveal the Molecular Mechanisms Underlying Hypoglycemic Nutrient Metabolites Biosynthesis in *Cyclocarya paliurus* Leaves During Different Harvest Stages

**DOI:** 10.3389/fnut.2022.851569

**Published:** 2022-02-28

**Authors:** Xuehai Zheng, Huibao Xiao, Jiannan Chen, Jinmao Zhu, Yajuan Fu, Songying Ouyang, Youqiang Chen, Duo Chen, Jingqian Su, Ting Xue

**Affiliations:** The Public Service Platform for Industrialization Development Technology of Marine Biological Medicine and Products of the State Oceanic Administration, Fujian Key Laboratory of Special Marine Bioresource Sustainable Utilization, Southern Institute of Oceanography, Key Laboratory of Developmental and Neural Biology, College of Life Sciences, Fujian Normal University, Fuzhou, China

**Keywords:** *Cyclocarya paliurus*, metabolome, whole-transcriptome, WGCNA, α-amylase

## Abstract

*Cyclocarya paliurus*, a well-known nutrient and beverage plant, is under development for use in functional health care products best and natural and organic foods. We hypothesis that the composition and metabolic accumulation of hypoglycemic nutrient metabolites exhibit significant differences depending on harvest time. Therefore, it is of great significance to establish the best harvest time for *C. paliurus* leaves for the further development of healthy teas and other products. However, the detail compositions and molecular mechanisms of nutrients biosynthesis in *C. paliurus* leaves during different harvest stages remain largely unclear. Metabolome analysis showed that a suitable leaf-harvesting strategy for *C. paliurus* could be in September or October each year due to the high content of hypoglycemic nutrient metabolites. We found that two of the seven differentially accumulated phenolic acid metabolites have a relatively good inhibitory effect on α-amylase, indicating that they may play a role in the hypoglycemic function. Combined analysis of coexpression, ceRNA network, and weighted gene correlation network analysis (WGCNA) showed that several genes or transcription factors (TFs) in three modules correlated highly with hypoglycemic nutrient metabolites, including *CpPMM, CpMan, CpFK, CpSUS, CpbglX, Cp4CL, CpHCT*, and *CpWRKY1*. These findings help in the understanding of the molecular mechanisms and regulatory networks of the hypoglycemic nutrient metabolites in *C. paliurus* leaves which are dependent on harvest time and provide theoretical guidance in the development of functional health care products and foods from *C. paliurus*.

## Highlights

- A suitable leaf-harvesting strategy for *C. paliurus* could be in September or October.
- Neochlorogenic acid and vanillic acid have a relatively good inhibitory effect on α-amylase.
- A total of 15,951 DE-mRNAs, 722 DE-miRNAs, 2,025 DE-lncRNAs, and 12 DE-circRNAs were identified.
- Multi-omics analysis showed that several genes or transcription factors (TFs) in three modules correlated highly with hypoglycemic metabolites.

## Introduction

*Cyclocarya paliurus* (Batal.) Iljinskaja, also known as the money tree, belongs to the family Juglandaceae and is one of the endangered medicinal plants protected by the state (1–4). *Cyclocarya paliurus* is a tall and fast-growing deciduous tree 10–30 m high that is widely distributed in the subtropical provinces of China (5). Studies reported that the leaves of *C. paliurus* are rich in secondary metabolites (such as flavonoids, triterpenoids, organic acids, phenolic acids, and polysaccharides), which have various pharmacological and healthy nutrient activities, including regulating blood sugar, lowering blood lipids, strengthening body immunity, antioxidation, antiaging, and anticancer activities (6–13). The high economic value of the biologically active nutrient substances in this plant is arousing increasing interest in the development of healthy foods and drugs. Healthy teas made from *C. paliurus* leaves have been promoted and welcomed by consumers, especially in diabetic consumers (lowering blood sugar), including one health tea derived from *C. paliurus* which has been e approved by the CFDA in China (14–16). Therefore, the development of related functional health care nutrient products or foods using *C. paliurus* leaves as raw materials has important research value and potential market prospects.

Young leaves are generally picked as raw material for tea production in China in consideration of consumers' preferences (flavor and taste), leading to a decrease in the leaf yield and thereby affecting commercial applications (17). To improve the resource utilization of *C. paliurus* leaves, multiomics have been used to determine the variation rules and the synthesis pathways of hypoglycemic nutrient metabolites in leaves at different maturities (18). Previous studies have shown that mixing leaves at three different stages of maturity was considered an optimal harvesting strategy based on consumer preferences and production costs (18). Additionally, the middle leaf is an ideal material for healthy tea production due to its high accumulation of secondary metabolites (18). The natural environment and climate can also greatly influence the metabolite profile in *C. paliurus* leaves, thereby affecting tea quality. Harvest time is an important factor to be considered in the development of healthy teas using these medicinal and bioactive ingredients. To date, there have been no reports describing the composition changes and metabolic accumulation of hypoglycemic nutrient metabolites in response to harvest time variation. In the production practices of *C. paliurus* tea, producers usually harvest leaves in the spring and autumn to produce healthy tea, which is similar to the process of traditional tea production. However, given the biological characteristics of *C. paliurus*, autumn leaves (August, September, October, and November) have become the main raw material for the production of tea due to there being very few leaves produced in spring. This suggests that *C. paliurus* leaves harvested in autumn may have a positive effect on tea quality.

In our previous study, we reported the genome assembly of *C. paliurus* (~634.90 Mb), providing a valuable resource for understanding the accumulation of hypoglycemic nutrient metabolites in *C. paliurus* leaves at different harvest times in autumn (18). In this study, to detect the changes in hypoglycemic metabolites in *C. paliurus* leaves at different harvest times (August, September, October, and November), as well as conducted a widely targeted metabolomic analysis using the UPLC–MS/MS detection platform. Additionally, an alpha-amylase inhibitory activity experiment was carried out to validate the hypoglycemic function of differentially accumulated hypoglycemic metabolites. To further understand the biosynthesis pathways and regulatory networks of the accumulation of hypoglycemic metabolites in *C. paliurus* leaves at different harvest times, coexpression, ceRNA network, and WGCNA were combined to mine the candidate genes involved in the accumulation and regulation of hypoglycemic nutrient metabolites, especially terpenoids, flavonoids, and polysaccharides. These findings will provide theoretical guidance for the development and utilization of functional health care products and foods derived from *C. paliurus*.

## Materials and Methods

### Materials

**Figure 1:**
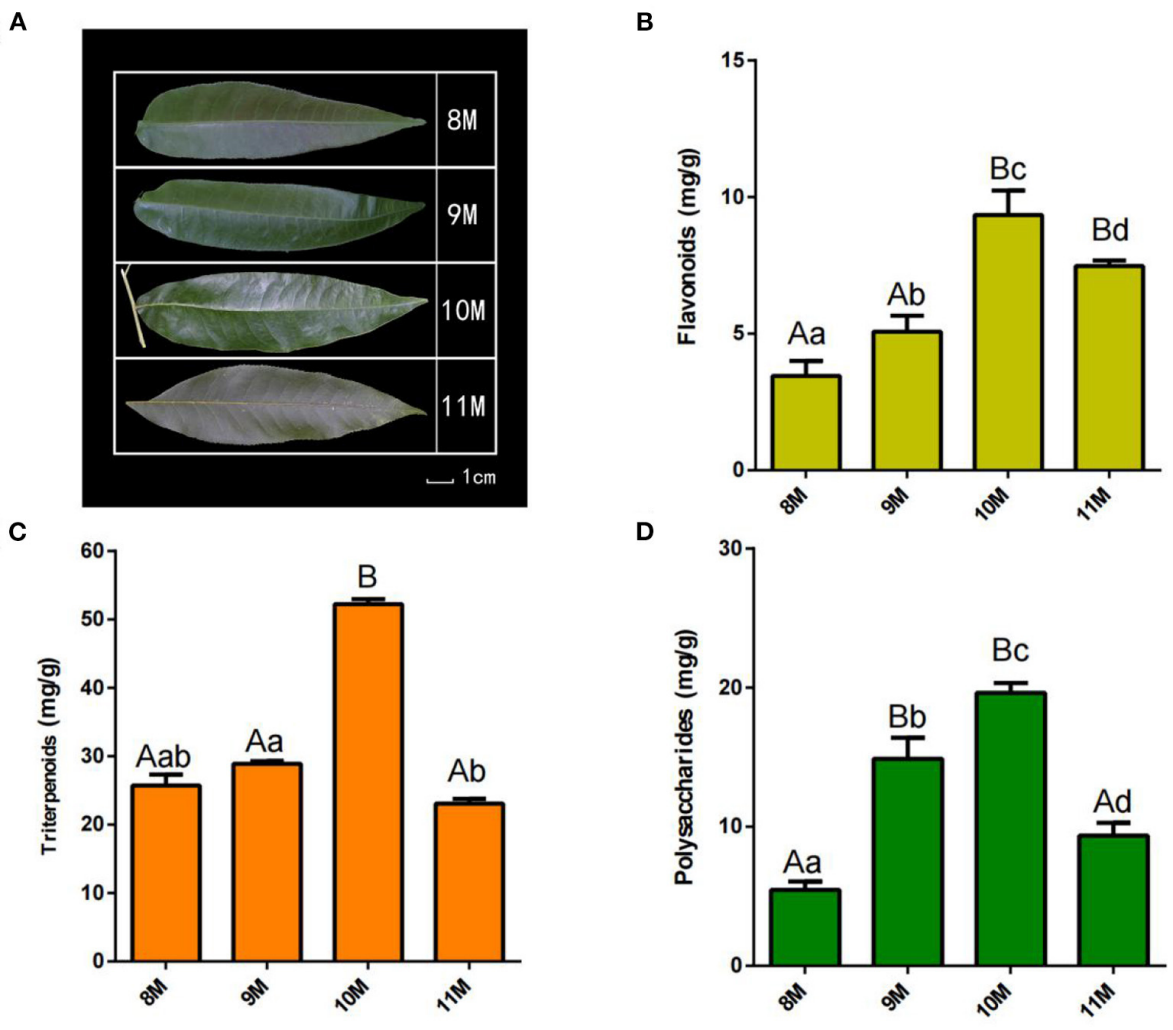
Phenotype and physiological-biochemical indices of *C. paliurus* leaves. **(A)** Morphological observation of *C. paliurus* leaves from different harvest times. **(B)** Total triterpenoids content. **(C)** Total polysaccharides content. **(D)** Total flavonoids content. Bar = 1 cm. Error bars indicate standard deviation. Capital letters and small letters indicate that significance is at 0.01 or 0.05 level.

*Cyclocarya paliurus* leaf samples from the same maturity (middle leaves) at different growth periods were collected from August 2020 to November 2020. The experiments were conducted on the middle leaves at each stage (8 M, harvested in August; 9 M, harvested in September; 10 M, harvested in October; and 11 M, harvested in November) (Figure 1A). The leaves were collected from the same three trees at different time periods with three biological replicates, and each tree was considered one biological repeat. Leaf samples were quickly frozen in liquid nitrogen and stored in a −80°C freezer for multiomics sequencing and analysis. Alpha-amylase (Cat# 8750) and α-amylase activity kits (Cat# BC0615) were purchased from Solarbio (Beijing, China). Neochlorogenic acid and vanillic acid assay kits were purchased from MedChemExpress (Shanghai, China).

### Metabolite Profiling

Total polysaccharides, triterpenoids, and flavonoids content were determined by using UV-vis spectrophotometry according to the Chinese industry standards of SN/T4260-2015, NY/T3676-2020, and SN/T4592-2016, respectively (Supplementary Figure S1). The detection and quantitative analysis of hypoglycemic nutrient metabolites in the samples were performed by Metware (http://www.metware.cn/) based on comparison with a self-built database of purified metabolite standards and public database. Briefly, the freeze-dried leaves were crushed by a grinding mill (MM 400, Retsch) at 30 Hz for 1.5 min. One hundred milligrams of powder was dissolved in 1.2 mL of 70% methanol, vortexed for 30 s, and placed at 4°C overnight. The supernatant was obtained by centrifugation (12,000 rpm, 10 min), filtered through a 0.22-μm membrane, and then analyzed using a UPLC/MS/MS system (19). The operating conditions of UPLC were as follows: column, Agilent SB-C18 column (1.8 μm, 2.1 × 100 mm); mobile solvent A (0.1% formic acid in water) and mobile solvent B (0.1% formic acid in acetonitrile); flow rate, 0.35 mL/min; injection volume, 4 μL. The operating parameters of the MS/MS were as follows: ion spray voltage, 5,500 V (+)/4,500 V (–); ion source gas, gas I (50 psi), gas II (60 psi), and curtain gas (25 psi); and ion source temperature, 550°C. All metabolites were identified qualitatively by MetWare database (MWDB), and isotope signals, repeated signals containing K^+^ ions, Na^+^ ions and ${\text{NH}}_{\text{4}}^{\text{+}}$ ions, as well as repeated signals of fragments of other substances with larger molecular weights were removed during analysis based on the secondary spectrum information. Metabolites were quantified by multiple reaction monitoring (MRM) using triple quadrupole mass spectrometry. After obtaining the metabolite spectrum analysis data of different samples, the peak area integration was performed on the mass spectrum peaks of all substances, and the integral correction was performed on the mass spectrum peaks of the same metabolite in different samples (20). Mass spectrum data was conducted with Analyst software (version 1.6.1) (21). The characteristic ions of each substance were screened by triple quadrupole, and the signal intensity of the characteristic ions was obtained in the detector. The mass spectrometry data was analyzed using MultiaQuant software, and the chromatographic peaks were integrated and corrected. The peak area of each chromatographic peak represents the relative content of the corresponding substance. Principal component analysis (PCA) was carried out by statistics function prcomp within R (www.r-project.org) with the parameter scale = True, and hierarchical cluster analysis (HCA) was performed by R package pheatmap. Orthogonal partial least squares-discriminant analysis (OPLS-DA) model was established by using function OPLSR.Anal within R package MetaboAnalystR (22, 23). Significantly regulated metabolites between groups were determined (log2-fold change ≥ 2 and variable importance in project ≥ 1) and then enriched using the KEGG database.

### Alpha-Amylase Inhibitory Activity Experiment

Alpha-amylase (EC 3.2.1.1) and α-glucosidase (EC 3.2.1.20) belong to glycosidases (EC 3.2.1) are involved in hydrolysis of starch into sugars and disaccharides, and inhibition of these enzymes activity can effectively lower the blood glucose level (24). Acarbose, as a weak and low-selective inhibitor, is the dominant drug in the field of diabetes by slowing down the absorption of glucose through the inhibition of α-amylase, howbeit with serious side effects (25). Inhibition of α-amylase activity provide an essential way for the treatment of type 2 diabetes mellitus (T2DM), which can be used as the basis for the development of new hypoglycemic drugs in the future (26). To detect the α-amylase inhibition activity, a mixture of 37.5 μL of α-amylase solution (0.1 mg/mL) and 5.0 μL of α-amylase inhibitor (neochlorogenic acid or vanillic acid) was first incubated at 40°C for 5 min, and then 75 μL of solution I (Cat#BC0615, Solarbio) was added. The reaction was stopped by heating for 10 min at 100°C after the addition of 37.5 μL of solution II (Cat#BC0615, Solarbio). The reaction solution was cooled to room temperature and then measured at 540 nm using a microplate reader (OPTIMA S/N413-3915). Acarbose and boiled α-amylase solutions were used as the positive and blank controls, respectively. A control reaction was used, in which the α-amylase solution was replaced with buffer solution. A negative reaction was used, in which the inhibitor was replaced with the buffer solution. Inhibitory concentrations of 50% (IC_50_) were analyzed by GraphPad Prism using the specific calculation formula (Y = Bottom + (Top-Bottom)/(1 + 10^∧^((LogIC_50_-X)^*^HillSlope))). The inhibition percentage of α-amylase was calculated by the following formula:


$$\begin{array}{l} \text{α}-\text{amylase inhibition percentage}\left(\text{\%}\right)\\ \;=\left[1-\left({\text{A}}_{3}-{\text{A}}_{4}\right)/\left({\text{A}}_{1}-{\text{A}}_{2}\right)\right]\times 100\text{\%}, \end{array}$$

where A_1_ = activity with negative control (without inhibitor), A_2_ = activity with blank control (with boiled α-amylase solution), A_3_ = activity with inhibitor (acarbose, neochlorogenic acid, or vanillic acid), and A_4_ = activity with control reaction (without α-amylase solution). One unit of enzyme inhibition was expressed by the weight of the IC_50_ value per milliliter.

### Whole Transcriptome Sequencing and Analysis

The preparation of total RNA was completed with the RNeasy plant mini kit (Tiangen Bio, Beijing, China). Construction of libraries for the rRNA-depleted stranded RNA-seq and small RNA was carried out with the TruSeq Stranded Total RNA Prep kit (Illumina, San Diego, USA) and the TruSeq® Small RNA sample preparation guide (Illumina, San Diego, CA), respectively. HISAT2 was used for aligning trimmed sequences to the reference genome (27). StringTie (version 1.3.1) was used to calculate the FPKM values of lncRNAs and mRNAs (28), miRNAs were detected using mirDeep2 (version 2.0.5), and miRNA expression was calculated from miRBase. TargetFinder (version 1.6) was used to predict miRNA target genes (29). CIRI (version 1.1.1) was used to identify and annotate the *de novo* circRNAs (30). Differential expression analysis of RNAs was performed (fold change ≥2.0, *p-*value < 0.05, and FDR <0.05) and then enriched by KEGG (31).

### Coexpression and ceRNA Network Analysis

Coexpression analysis was performed by a threshold of PCC ≥ 0.9 and a *p*-value < 0.01 (32–35). The ceRNA relationship pairs were obtained through the targeting relationship of miRNAs based on the number of miRNAs > 5, FDR ≤ 0.01, and a *p*-value < 0.01. Based on the ceRNA results, the relationship pairs of all differences of lncRNA-miRNA-mRNA and circRNA-mRNA-mRNA were obtained and visualized using Cytoscape (version 3.7.2) (36).

### WGCNA Analysis

The transcriptome data were normalized using the normalize quantiles function in the R software package, and all differentially expressed genes (DEGs) between samples were used for further WGNCA analysis (35). The eigengenes of each module were calculated, and then cluster analysis was performed on the modules. WGCNA was performed as follows: minimum module size, 30; soft power, 13; and merge cut height, 0.15 (Supplementary Figure S2). Eigengene values of modules related to polysaccharides, triterpenoids, and flavonoids were calculated. The hub genes within significant modules were considered with a coefficient > 0.6 and a *p*-value < 0.05. All genes and module eigengene genes in the module were analyzed by expression level, GO, and KEGG analyses. Cytoscape (version 3.7.2) was used to visualize the coexpression network of ME genes in the module of interest (36).

### Quantitative Real-Time PCR

Total RNA was completed by using the TransZol Up Plus RNA Kit (Transgen Biotech, Beijing). First-strand cDNA was synthesized by TransScript One-Step gDNA Removal and cDNA Synthesis SuperMix (Transgen Biotech, Beijing) followed by analysis using the SYBR PrimeScript RT–PCR kit (Takara, Beijing). The specific primers for qRT–PCR were designed using Primer 5.0 (Supplementary Table S1). The relative level of mRNA expression was calculated according to the 2^−Δ*ΔCt*^ method and GAPDH was used as the reference gene (37).

## Results

### Hypoglycemic Nutrient Metabolite Profiles of *C. paliurus* Leaves During Different Harvest Stages

**Figure 2:**
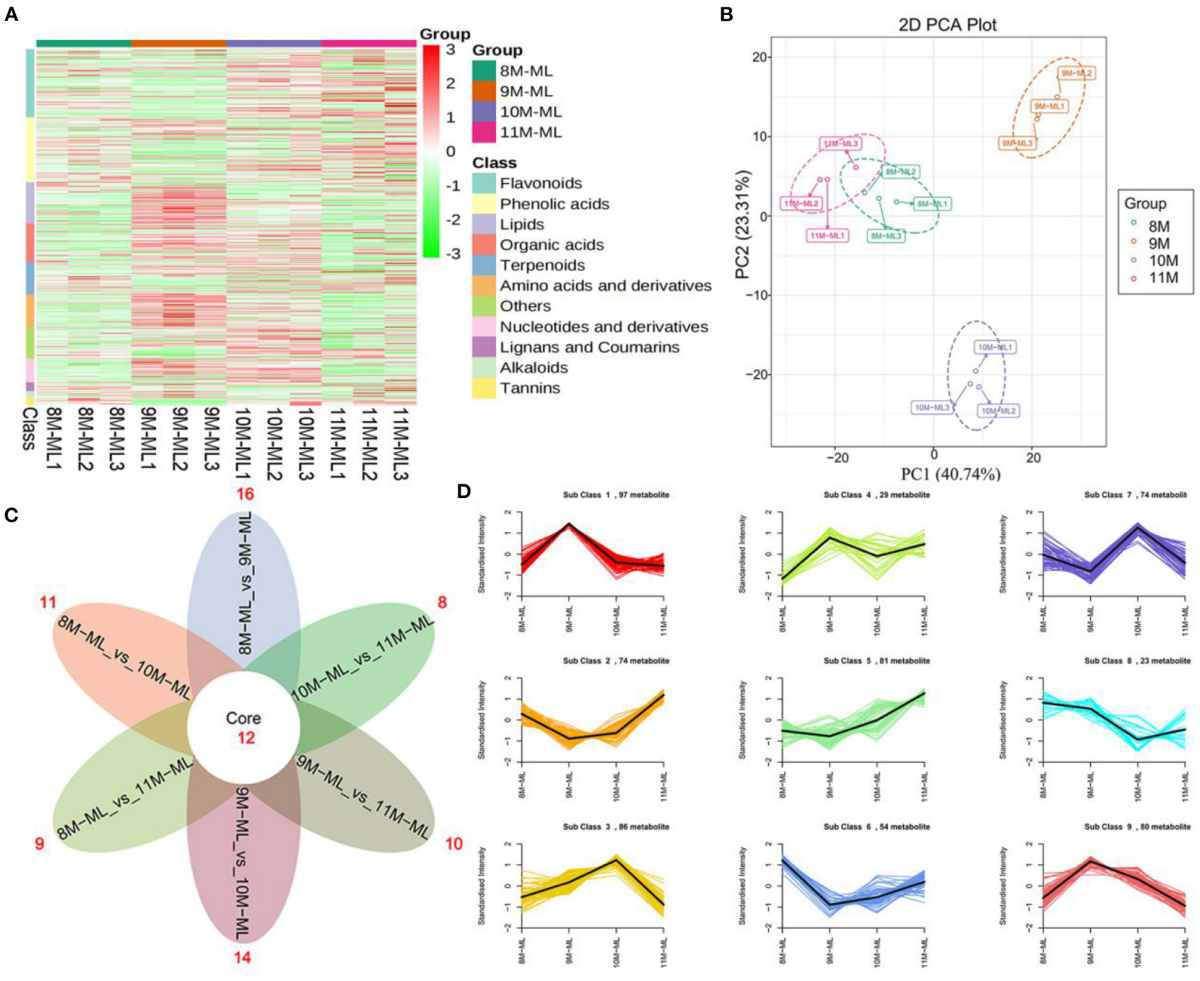
Metabolome profiles of *C. paliurus* leaves from different harvest times. **(A)** HCA of metabolites in 8–11 M. **(B)** PCA of 8–11 M. **(C)** Venn diagram of DAMs among 8M_vs_9 M, 8M_vs_10 M, 8M_vs_11 M, 9M_vs_10 M, 9M_vs_11 M, and 10M_vs_11 M. **(D)** K-means analysis of DAM *C. paliurus* leaves from different harvest times.

**Figure 3:**
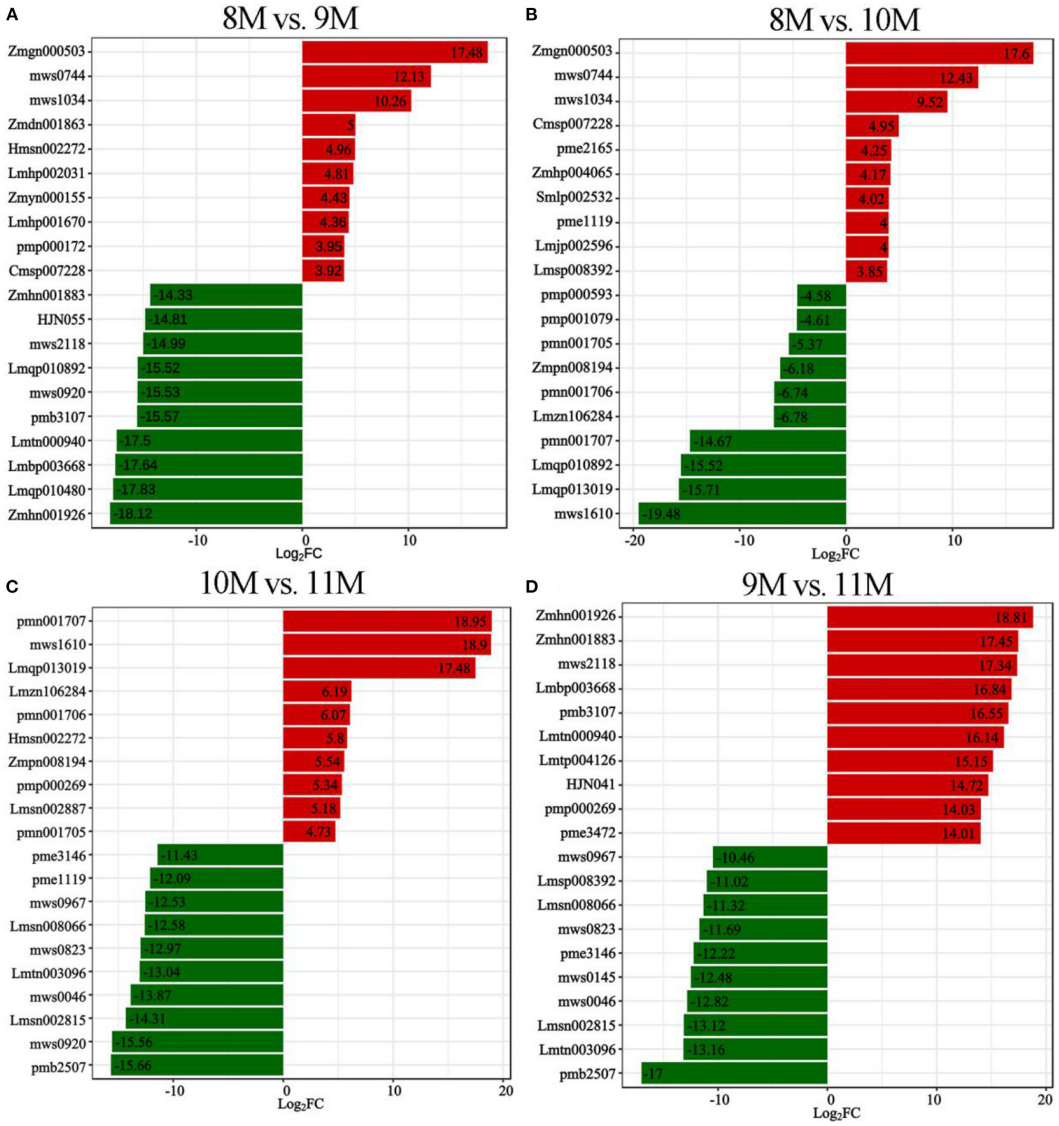
Differential metabolite expression analysis. Top 20-fold changes in DAMs in the 8M_vs_9 M **(A)**, 8M_vs_10 M **(B)**, 10M_vs_11 M **(C)**, and 9M_vs_11 M **(D)** groups. X-axis represents the log_2_FC of DAMs, and Y-axis represents the different DAMs. Red and green represent up- and down-regulated DAMs, respectively.

To identify the major metabolites in *C. paliurus* leaves at different harvest stages, we determined the total polysaccharides, triterpenoids, and flavonoids content (Figures 1B–D; Supplementary Tables S2–S4). The results showed that the total flavonoids content increased significantly during the development of *C. paliurus* leaves: from 4.09 mg/g in August (8 M) to 9.34 mg/g in October (10 M) (Figure 1B). The total triterpenoids content of 10 M (51.89 mg/g) was 1.90-, 1.80-, and 2.19-fold higher than that of the 8 M (27.33 mg/g), 9 M (28.79 mg/g), and 11 M (23.66 mg/g) groups, respectively (Figure 1C). In addition, the total polysaccharides content in *C. paliurus* leaves reached its maximum in the 10 M group but started to decrease significantly in the 11 M group (Figure 1D). Therefore, the findings confirmed that October may be conducive to the accumulation of hypoglycemic nutrient metabolites. To elucidate the potential chemical basis for the nutritional quality at different harvest stages, widely targeted metabolomic analysis was conducted using the UPLC–MS/MS detection platform. PCA and HCA analysis indicated that the metabolic profiles in *C. paliurus* leaves at different harvest stages exhibited a significant difference (Figures 2A,B). Based on the results from the volcano plot, among the 751 metabolites detected there were: 363 differentially accumulated metabolites (DAMs) (203 up- and 160 downregulated) between the 8 M and 9 M, 286 DAMs (187 up- and 99 downregulated) between the 8 M and 10 M, 216 DAMs (106 up- and 110 downregulated) between the 8 and 11 M, 295 DAMs (182 up- and 113 downregulated) between the 9 and 10 M, 373 DAMs (170 up- and 203 downregulated) between the 9 and 11 M, and 323 DAMs (124 up- and 199 downregulated) between the 10 and 11 M (fold change ≥ 2 and VIP ≥ 1) (Supplementary Figure S3; Supplementary Table S5). The Venn diagram analysis revealed that 12 DAMs were shared among all samples, whereas 16, 11, 9, 14, 10, and 8 different DAMs were unique to 8M_vs_9 M, 8M_vs_10 M, 8M_vs_11 M, 9M_vs_10 M, 9M_vs_11 M, and 10M_vs_11 M, respectively, indicating that the quality changes of *C. paliurus* leaves may be related to the regulation of these unique DAMs in multiple pathways (Figure 2C). Additionally, these DAMs were classified into nine groups, with the largest number of metabolites falling under flavonoids (124, 20.74%), followed by phenolic acids (100, 16.72%), lipids (77, 12.88%), amino acids, and derivatives (61, 10.20%), organic acids (59, 9.87%), and terpenoids (54, 9.03%) (Figure 2D). The contents of phenolic acids (vanillic acid, 1.17-fold increase; 4-hydroxybenzoic acid, 1.29-fold increase; anthranilic acid, 1.59-fold increase; caffeic acid, 2.23-fold increase) and flavonoids (dihydromyricetin, 12.12-fold increase; kaempferol-3-O-neohesperidoside, 1.37-fold increase; quercetin-3-O-sambubioside, 2.11-fold increase) were significantly higher in the 9 M group than in the 8 M group (Figure 3A; Supplementary Table S6). In the comparison of 8 vs. 10 M, most secondary metabolites (2,3-dihydroxy-3-methylbutanoic acid, 17.60-fold increase; dihydromyricetin, 12.43-fold increase; cyclocaric acid B, 3.22-fold increase; cyclocaric acid A, 2.28-fold increase; neochlorogenic acid, 1.06) were upregulated in 10 M compared to 8 M (Figure 3B; Supplementary Table S6). In the comparison of 10 M vs. 11 M, the metabolite profiles associated with flavonoids (tricetin, 15.56-fold decrease; kaempferol-3-O-rutinoside, 14.31-fold decrease), phenolic acids (2-methoxybenzoic acid, 10.98-fold decrease; acetovanillone, 10.18-fold decrease; neochlorogenic acid, 1.33-fold decrease), and nucleotides and derivatives (inosine, 12.09-fold decrease; 2-deoxyribose-1-phosphate, 15.65-fold decrease) were significantly downregulated in the 11 M group compared with the 10 M group (Figure 3C; Supplementary Table S6). The number of DAMs between the 9 M and 11 M groups was greatest among these comparison groups, and the number of downregulated DAMs (203) was 1.19-fold higher than that of upregulated DAMs (170), indicating that the quality of *C. paliurus* leaves may decline from the 9 M to the 11 M stage (Figure 3D; Supplementary Table S6). Considering the accumulation of metabolites, a suitable harvesting strategy for *C. paliurus* leaves could be in September or October each year.

### Alpha-Amylase Inhibitory Activity

**Figure 4:**
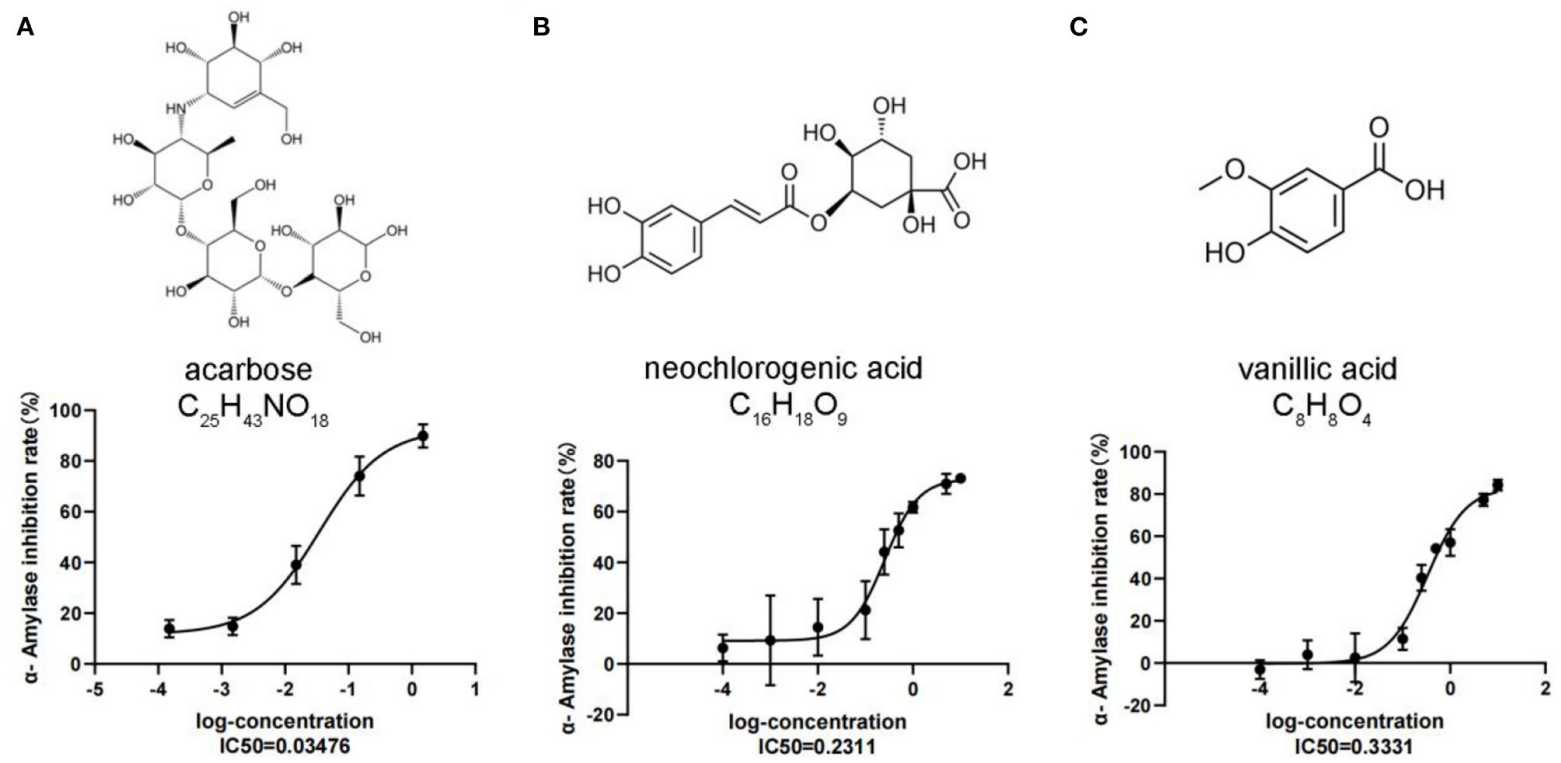
Alpha-amylase inhibitory assay of DAMs. **(A)** Acarbose. **(B)** Neochlorogenic acid. **(C)** Vanillic acid. Each value represents the mean ± SD, and error bars represent significant differences (*n* = 3, *P* < 0.05).

To confirm the metabolomic results and also to develop potential alpha-amylase inhibitors from *C. paliurus* leaves, an α-amylase inhibitory activity assay was conducted on seven differentially accumulated phenolic acid metabolites selected based on the metabolic changes and differential analysis, including vanillic acid, 4-hydroxybenzoic acid, anthranilic acid, caffeic acid, 2-methoxybenzoic acid, acetovanillone, and neochlorogenic acid. The α-amylase inhibitory activity results showed that neochlorogenic acid, vanillic acid, and acarbose reduced the stability of the α-1,4-glucoside bond with IC_50_ values of 0.23, 0.33, and 0.03 mM, respectively. We also found that the highest inhibition of neochlorogenic acid and vanillic acid was 61.74 and 57.03% at a concentration of 1.0 mM, which was lower than that of acarbose at 1.0 mM (85.67%) (Figure 4). The results showed that neochlorogenic acid and vanillic acid have a relatively good inhibitory effect on α-amylase, indicating that they may play a role in hypoglycemic functions.

### Differential Expression Analysis of lncRNAs, miRNAs, mRNAs, and circRNAs

**Figure 5:**
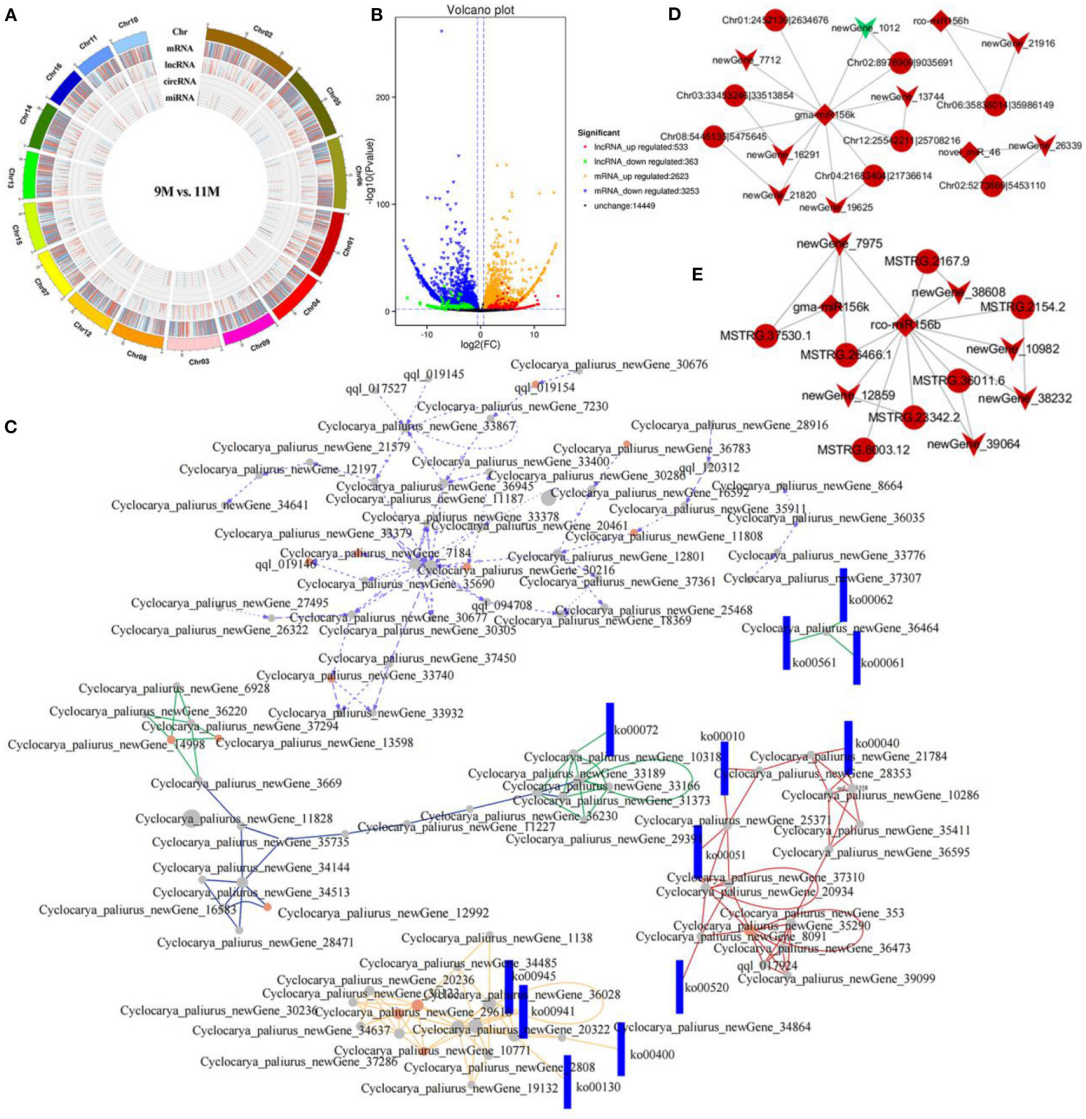
Coexpression and ceRNA network analysis. **(A)** Circos diagram of DE-RNAs in the 9M_vs_11 M group. **(B)** Volcano diagram of the DE-lncRNAs and DE-mRNAs in the 9M_vs_11 M group. **(C)** CeRNA networks in the 10M_vs_11 M group. Dots and rectangles represent genes and pathways, respectively. The colors of different lines represent relationships from different paths. **(D)** CircRNA-miRNA-mRNA network. Circles, diamonds, and arrows represent circRNA, miRNA, and mRNA, respectively. **(E)** LncRNA-miRNA-mRNA network. Circles, diamonds, and arrows represent lncRNAs, miRNAs, and mRNAs, respectively.

To identify the critical genes associated with hypoglycemic nutrient metabolites in *C. paliurus* leaves, the whole transcriptome was further analyzed. A total of 8,805 lncRNAs, 3,2164 mRNAs, 899 circRNAs, and 496 miRNAs (43 known miRNAs, 453 novel miRNAs, and 4,125 target genes) were identified in the 8–11 M groups, respectively (Figure 5A; Supplementary Figure S4A). Of those, 21,411 (84.28%) of 25,405 new genes were successfully annotated to nine public databases, including COG (6,654, 26.19%), KEGG (14,938, 58.79%), GO (17,461, 68.73%), KOG (11,800, 46.45%), Pfam (16,626, 65.44%), SwissProt (15,067, 59.31%), TrEMBL (21,343, 84.01%), eggNOG (18,139, 71.40%), and NR (21,313, 83.89%) (Supplementary Figure S4B; Supplementary Table S7). Based on the expression profiles and differential analysis, a total of 15,951 differentially expressed (DE) mRNAs were identified, among which 7,110 and 8,841 DE-mRNAs showed upregulation and downregulation, respectively (Supplementary Figure S4C). Among these DE-mRNAs, the largest number of DEGs fell under 8M_vs_11 M (2,828 upregulated and 4,064 downregulated), followed by 9M_vs_11 M (2,623 upregulated and 3,253 downregulated), and 8M_vs_9 M (1,226 upregulated and 1,184 downregulated), and the number of downregulated genes (4,064) was 1.44-fold higher than that of upregulated genes (4,064) from the 8M_vs_11 M group (Figure 5B; Supplementary Figure S5). Enrichment analysis showed that these DE-mRNAs were mainly associated with GO:0006952~defense response, GO:0045490~pectin catabolic process, phenylpropanoid biosynthesis, plant hormone signal transduction, glucuronate interconversions, fructose metabolism, diterpenoid biosynthesis, and fatty acid degradation (Figure 5C). In the comparison of 8 vs. 11 M, most downregulated DEGs were enriched in the starch and sucrose pathway (*CpsacA*, newGene_11051; *CpSUS*, newGene_11769; *CpGH12*, newGene_18867; *CpHK*, newGene_16922), amino sugar and nucleotide sugar metabolism (*CpgalE*, newGene_7290; *CpglgC*, newGene_2262; *CpUSP*, newGene_15611; *CpPMM*, newGene_21902), and fructose and mannose metabolism (*CpPFP*, newGene_9652; *CpGMPP*, newGene_5583; *CpMAN*, newGene_11578) (Supplementary Figure S6A). Notable decreases in transcriptome expression from the 9 M to 11 M group included terpenoid biosynthesis (*CpCYP76F14*, newGene_34701; *CpLUS*, newGene_10624; *CpNES1*, newGene_8992; *CpKAO*, newGene_27209) and flavonoid biosynthesis (*CpCYP3A*, newGene_20322; *CpANR*, newGene_20574; *CpHCT*, newGene_22094; *CpDFR*, newGene_25488; *CpANS*, newGene_35209) (Supplementary Figure S6B). We hypothesize that most genes involved in secondary metabolites are significantly downregulated in November collected *C. paliurus* leaves compared with those collected in August and September. For miRNAs, 3,703 (89.77%) of 4,125 target genes were successfully annotated to nine public databases, including COG (1,460, 35.39%), KEGG (2,760, 66.91%), GO (3,177, 77.02%), KOG (2,112, 51.20%), Pfam (3,051, 73.96%), SwissProt (2,828, 68.56%), eggNOG (3,275, 79.39%), and NR (3,698, 89.65%) (Supplementary Figure S7; Supplementary Table S8). Of these, 722 DE-miRNAs were identified by pairwise comparison, of which 404 were upregulated and 318 were downregulated (Supplementary Table S9). Enrichment analysis of DE-miRNA target genes showed that they were mainly enriched in plant–pathogen interactions, plant hormone signal transduction, glycosphingolipid biosynthesis, terpenoid biosynthesis, starch and sucrose metabolism, and phenylpropanoid biosynthesis (Supplementary Figure S8). Additionally, we also obtained 12 DE-circRNAs, including 9 upregulated and 3 downregulated circRNAs. For example, DE-circRNA Chr10:10:9049396|9051972 was upregulated with *CpNAC62* (newGene_16052), which is involved in the defense response; DE-circRNA Chr03:22680395|22837830 was positively correlated with *CpGME* (newGene_37112), which is related to amino sugar and nucleotide sugar metabolism (Supplementary Figure S9).

### Coexpression and ceRNA Network Analysis

A coexpression network was constructed, resulting in 1,048,575 lncRNA-mRNA, 296,770 circRNA-mRNA, 189,105 circRNA-lncRNA, and 589 miRNA-circRNA networks. A ceRNA network was generated with 76 nodes based on the coexpression relationship, including 16 lncRNAs, 32 circRNAs, and 28 mRNAs. Enrichment analysis implied that the relationship pairs from DE-ceRNAs mainly participated in alpha-linolenic acid metabolism, MAPK signaling pathway, phenylpropanoid biosynthesis, and diterpenoid biosynthesis (Supplementary Figure S10). The circRNA-miRNA-mRNA network indicated that gma-miR-156k, one of the key miRNAs, inhibited the expression of *CpPIP5K* (newGene_1012) by regulating Chr02:8978909|9035691. Moreover, circRNAs Chr08:5445135|5475645 and *CpMFP2* (newGene_21820) were positively correlated with gma-miR-156k, which is associated with alpha-linolenic acid metabolism (Figure 5D). The lncRNA-miRNA-mRNA network indicated that rco-miR156b established common connections with six lncRNAs and five mRNAs. LncRNA MSTRG.26466.1 acted as a sponge of rco-miR156b to regulate the target gene *CpGBE1* (newGene_7975), which is related to starch and sucrose metabolism (Figure 5E).

### Hub Gene Identification Involved in Hypoglycemic Nutrient Metabolites

**Figure 6:**
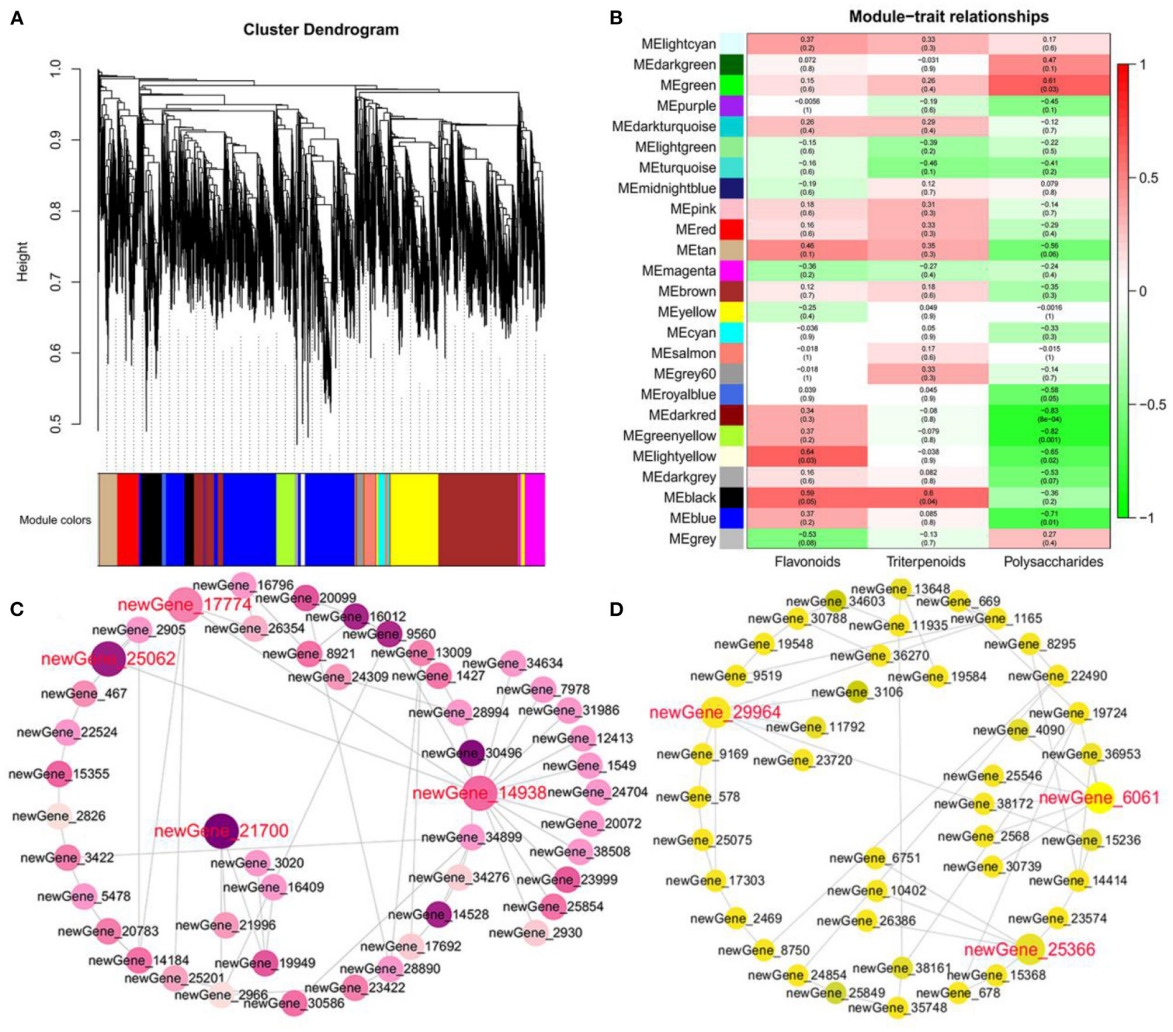
Hub gene identification involved in hypoglycemic nutrient metabolites. **(A)** Tree diagram of all DEGs based on a value of dissimilarity measure (1-TOM). **(B)** Gene modules associated with the content of triterpenoids, polysaccharides, and flavonoids. **(C)** Coexpression network revealing the hub genes related to polysaccharides in the green module. **(D)** Coexpression network revealing the hub genes related to flavonoids in the light yellow module.

To identify the hub genes associated with polysaccharides, triterpenoids, and flavonoids, we performed WGCNA for the RNA-seq data of 12 samples. We identified 25 modules (comprising of 24 coexpression modules and one module with uncorrelated genes), and the number of genes ranged from 35 to 2,938 (Figure 6A). Notably, the green module was found to have a significantly high association with polysaccharides (*r* = 0.61 and *p*-value = 0.03) (Figure 6B). Enrichment analysis showed that genes in the green module were significantly involved in galactose metabolism, sucrose metabolism, and fructose metabolism, including *CpPMM* (newGene_21902), *CpMan* (newGene_25062), *CpFK* (newGene_21700), *CpSUS* (newGene_17938), *CpTPS* (newGene_30013), *CpbglX* (newGene_17774), *CpGN1* (newGene_6921), *CpPYG* (newGene_13377), and *CpglgC* (newGene_2262) (Figure 6C; Supplementary Figure S11). Furthermore, we found that two modules, dark-red (*r* = −0.83 and *p*-value = 8E-4) and green-yellow (*r* = −0.82 and *p*-value = 0.001), were significantly and negatively correlated with polysaccharides (Figure 6B). *CpsucA* (newGene_35015), *CpsucB* (qql_045894), *CpENO* (qql_090117), and *CpCS* (qql_024905) in the green-yellow module were annotated to be associated with the citrate cycle and glycolysis (Supplementary Figure S12). We hypothesis that these genes can reduce the production of glucose and energy by playing an important negative regulatory role in polysaccharide metabolism. Another light-yellow module was detected, which tended to be involved in flavonoids (*r* = 0.64 and *p*-value = 0.03) (Figure 6B; Supplementary Figure S13). We captured two hub genes (*CpHCT*, newGene_24442; *Cp4CL*, newGene_25920), which shared 29 and 44 edges with other candidate genes. In addition, we also identified 17 TFs in the black module and found several TFs that are highly positively correlated with triterpenoids, including two MYB-related genes (newGene_2469, newGene_19602) and one WRKY gene (*CpWRKY1*, newGene_8245) (Figure 6D; Supplementary Figure S14). To further confirm the reliability of the RNA-Seq analysis, qRT–PCR was performed on five DEGs, including *CpFK, CpSUS, CpHCT, Cp4CL*, and *CpWRKY1*. The results of qRT–PCR were similar to the FPKM values from RNA-Seq, demonstrating the high reliability of the RNA-seq data (Supplementary Figure S15).

## Discussion

The leaves of *C. paliurus* are rich in antioxidant active substances, such as flavonoids, triterpenoids, polysaccharides, and phenolic acids, which are beneficial to human health, especially for people suffering from diabetes and hypertension (38, 39). There is an increased demand for the development and utilization of health care tea from *C. paliurus leaves* for commercial uses. Cultivation techniques of this species have been established, including soil and light, while leaf-harvesting strategies have not yet been carried out (40, 41). A suitable harvesting strategy for *C. paliurus* leaves is considered an important factor for the development of healthy tea using diverse biological activities. We found that October may be the most conducive month of harvest for the accumulation of polysaccharides, triterpenoids, and flavonoids. To detect the composition changes and metabolic accumulation of hypoglycemic nutrient metabolites in response to harvest time, a widely targeted metabolomic analysis was conducted using the UPLC/MS/MS detection platform. A total of 751 metabolites and 363 DAMs were identified, mainly flavonoids, phenolic acids, lipids, and terpenoids. The contents of phenolic acids (vanillic acid, 4-hydroxybenzoic acid, anthranilic acid, and caffeic acid) and flavonoids (kaempferol-3-O-neohesperidoside, dihydromyricetin, and quercetin-3-O-sambubioside) in the 9 M group were significantly higher than those in the 8 M group. We also found that the different members associated with flavonoids (tricetin, kaempferol-3-O-rutinoside) and phenolic acids (2-methoxybenzoic acid, acetovanillone, and neochlorogenic acid) were significantly downregulated in the 11 M group compared to the 10 M group. We found that most DAMs (flavonoids and phenolic acids) associated with the hypoglycemic function of *C. paliurus* leaves significantly accumulated in the 9 and 10 M groups and declined from the 10 M to the 11 M group, indicating that a suitable leaf-harvesting strategy for *C. paliurus* could be in September or October each year. T2DM is an expanding global health problem with a worldwide prevalence rate of 9.3% (463 million diabetic patients) in 2019, causing a huge crisis to health care systems around the world (42). Alpha-amylase is associated with the hydrolysis of starch into disaccharides, resulting in an increase in blood sugar levels (43, 44). Therefore, inhibition of α-amylase activity plays an important role in the treatment of T2DM. Phenolic acid is a mixed inhibitor of α-amylase and α-glucosidase, which can be used to delay the absorption of glucose in the small intestine (45). Ferulic acid, ρ-coumaric acid, and chlorogenic acid showed inhibitory effects on α-amylase at a concentration of 0.05%, resulting in inhibition rates of 36.60, 59.35, and 51.43%, respectively (46). We found that the highest inhibition of neochlorogenic acid and vanillic acid was 61.74 and 57.03%, respectively, at a concentration of 1.0 mM of the enzymes. The results showed that the α-amylase inhibitory effects of neochlorogenic acid and vanillic acid were significantly higher than those of the three phenolic acids reported above but were lower than that of acarbose (85.67%). An alpha-amylase inhibitor of acarbose is the dominant drug in the field of diabetes, but it also has some toxicity and side effects, such as diarrhea, abdominal pain, and abdominal discomfort (47). Therefore, it is necessary to search for α-amylase inhibitors in natural products. Leaves from *C. paliurus* were approved as a food for both medicinal and food use based on the results of safety and toxicity tests and can be developed for daily health tea. Neochlorogenic acid and vanillic acid from *C. paliurus* leaves have a relatively good inhibitory effect on α-amylase, which may be considered for subsequent development of novel glycosidase inhibitors in hypoglycemic function, providing target metabolites for breeding of *C. paliurus*.

The traditional view of gene regulation focused on mRNA until the discovery of numerous lncRNAs, miRNAs, and circRNAs, and these ncRNAs can regulate important mRNA and ceRNA regulatory networks in very complex regulatory processes (48). The whole-transcriptome data analysis found that a total of 15,951 DE-mRNAs, 722 DE-miRNAs, 2,025 DE-lncRNAs, and 12 DE-circRNAs were identified by pairwise comparison. Enrichment analysis showed that these DE-RNAs were enriched in starch and sucrose metabolism, terpenoid biosynthesis, plant hormone signal transduction, glycosphingolipid biosynthesis, phenylpropanoid biosynthesis, and fatty acid degradation. Previous studies have shown that most members of the gma-miR156 family are related to drought resistance and nutritional stress of plants, which is the control center of plant growth and development (49–51). Overexpression of gma-miR156b in soybean and wheat can significantly increase the number of branches and pods per plant (52). gma-miR156 plays a role in the development of *Phaseolus vulgaris* roots and nodules and participates in the nutritional stress response of *P. vulgaris* (53). The A ceRNA network was obtained with 76 nodes based on the coexpression relationship, including 16 lncRNAs, 32 circRNAs, and 28 mRNAs. Among them was gma-miR-156k which is positively associated with signaling, germination, and alpha-linolenic acid metabolism in plants by regulating circRNA and mRNA. These results indicated that gma-miR-156k is one of the key miRNAs which may play fundamental functions in secondary metabolite synthesis.

Flavonoids, triterpenoids, and polysaccharides are not only the main components in the leaves of *C. paliurus* for lowering blood sugar but also an important indicator that determines the quality of healthy tea (18). To better understand the biosynthesis pathway and regulatory mechanism of these hypoglycemic nutrient metabolites, WGCNA was combined to further reveal the candidate genes involved in the accumulation of flavonoids, triterpenoids, and polysaccharides. In this study, nine genes in the green module were found to have a significantly high association with polysaccharides, including *CpPMM*, which catalyzes the conversion of mannose-6-phosphate to mannose-1-phosphate (54), *CpMan*, which is related to fructose and mannose metabolism (55), *CpFK*, which mediates the fucose salvage pathway (56), and *CpSUS* and *CpbglX*, which participate in starch and sucrose metabolism (57). These results indicate that early biosynthesis genes related to the precursors (starch, sucrose, fructose, and mannose) of polysaccharide biosynthesis are key enzyme genes for controlling the accumulation of downstream polysaccharides. Moreover, we captured two hub genes in the light-yellow module, which shared 29 and 44 edges with other candidate genes and were highly correlated with flavonoids. For example, *Cp4CL*, which is an important branch point for directing metabolites to flavonoids (58), and *CpHCT*, which is associated with monolignol biosynthesis and phenylpropanoid metabolism (59, 60). Interestingly, several TFs were significantly correlated with triterpenoid biosynthesis in the black module, including two MYB-related genes and *CpWRKY1*. Studies have shown that MYB and WRKY are related to triterpenoid and phenolic acid biosynthesis in *Panax notoginseng* and *Prunella vulgaris* (61, 62). *WsWRKY1* regulates triterpenoid biosynthesis by binding W-box sequences in the promoters of squalene synthase and squalene epoxidase (63). *BpMYB21* can positively regulate the response to methyl-jasmonate in birch triterpenoid biosynthesis, playing a key role in triterpene production (61). These results indicated that related members of the MYB and WRKY gene families in the black module may affect the biosynthesis of triterpenoids and phenolic acids by regulating the flow of metabolic intermediates during the synthesis of triterpenoids and phenolic acids. These results will help us better understand the molecular mechanisms and regulatory networks of the hypoglycemic nutrient metabolites in *C. paliurus* leaves at different harvest times and provide theoretical guidance for the development of functional health care products and foods derived from *C. paliurus*.

## Data Availability Statement

The datasets presented in this study can be found in online repositories. The name of the repository and accession number can be found below: Genome Sequence Archive (GSA) in National Genomics Data Center (NGDC), China National Center for Bioinformation (CNCB)/Beijing Institute of Genomics (BIG), Chinese Academy of Sciences (CAS), https://ngdc.cncb.ac.cn/gsa/, CRA005839.

## Author Contributions

DC, JS, and TX designed and coordinated the entire project. HX, YF, SO, JS, and YC performed the collection and processing of samples. XZ, JC, DC, and TX performed the analyses of metabolome and whole-transcriptome. TX, JZ, SO, and YC participated in manuscript writing and revision. All authors read and approved the final manuscript.

## Funding

This work was supported by the Fujian Provincial Regional Development Project (2021N3005) and Special Sci-tech Team Commissioner of Fujian Province (T202005007 and T202005013).

## Conflict of Interest

The authors declare that the research was conducted in the absence of any commercial or financial relationships that could be construed as a potential conflict of interest.

## Publisher's Note

All claims expressed in this article are solely those of the authors and do not necessarily represent those of their affiliated organizations, or those of the publisher, the editors and the reviewers. Any product that may be evaluated in this article, or claim that may be made by its manufacturer, is not guaranteed or endorsed by the publisher.
