# Supplementary material for: Metabolome and Whole-Transcriptome Analyses Reveal the Molecular Mechanisms Underlying Hypoglycemic Nutrient Metabolites Biosynthesis in Cyclocarya paliurus Leaves During Different Harvest Stages
Source: Front Nutr. 2022 Feb 28;9:851569. doi: 10.3389/fnut.2022.851569 (PMC8919051; doi:10.3389/fnut.2022.851569)
Supplement: Supplementary file 1 [file Data_Sheet_1.doc]

**Supplementary Figures**

A B


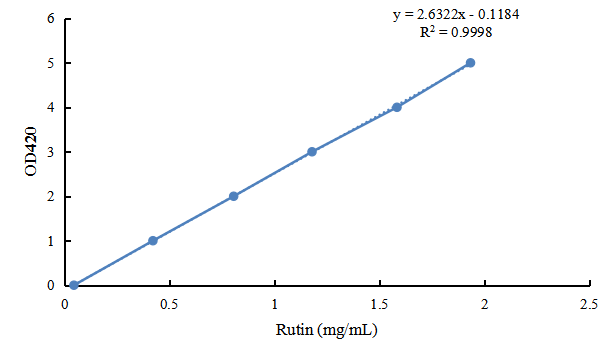

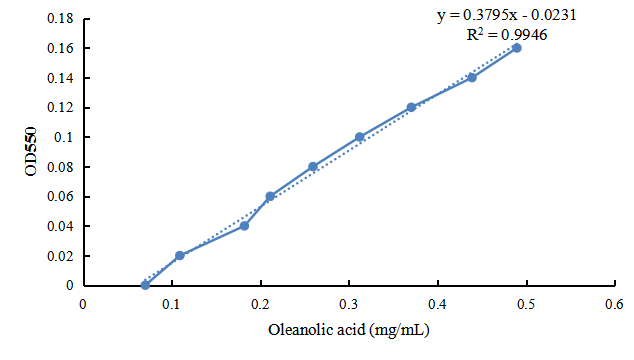
 C


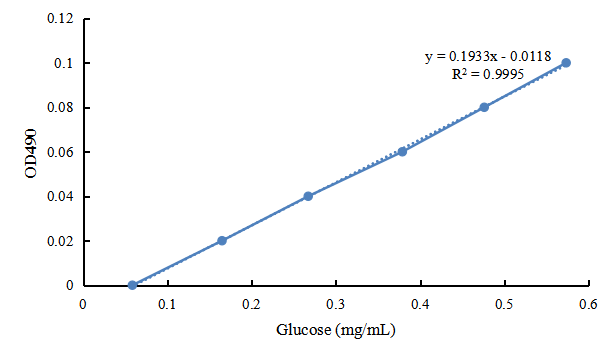


Fig. S1 Standard curve of flavonoids (A), triterpenoids (B), and polysaccharides (C).


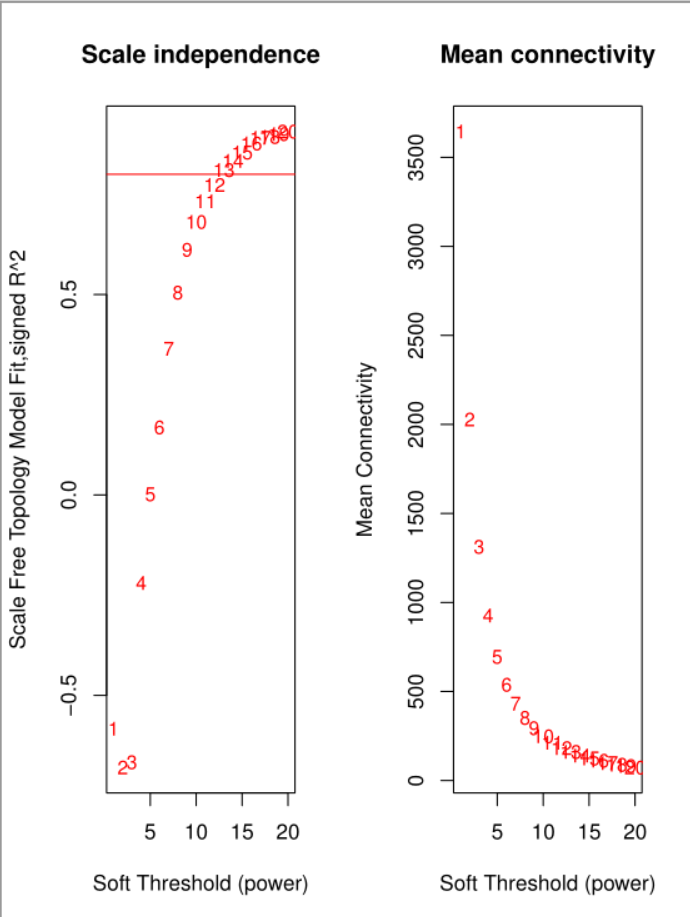


Fig. S2 A soft power of 13 for WGCNA analysis.

A B


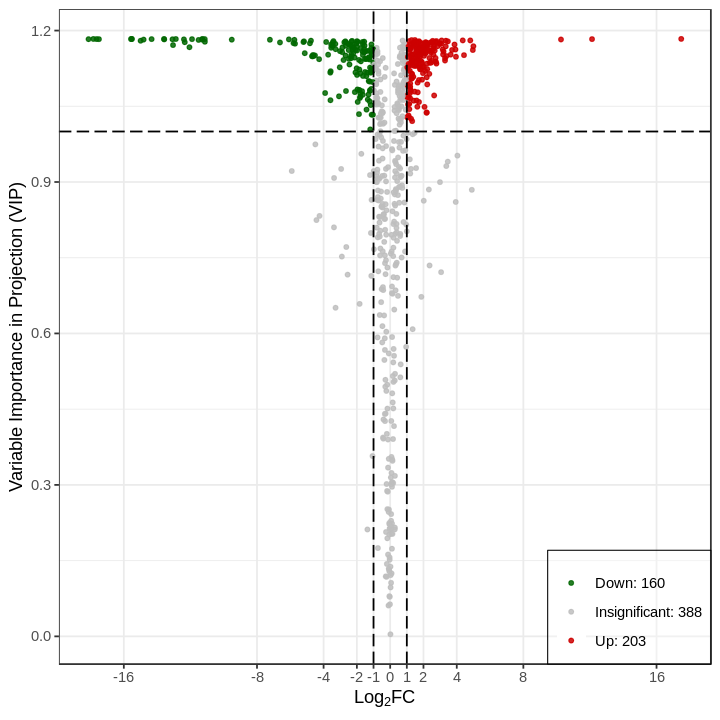

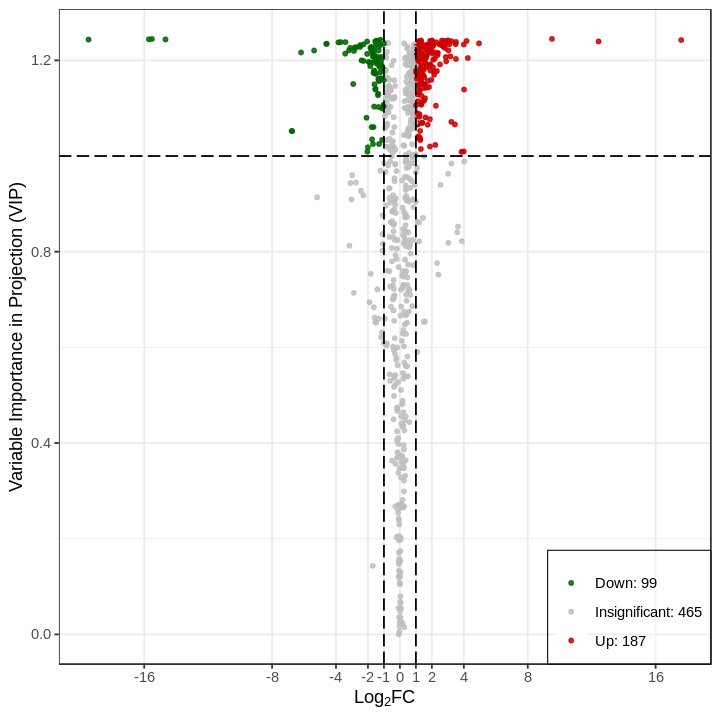


C D


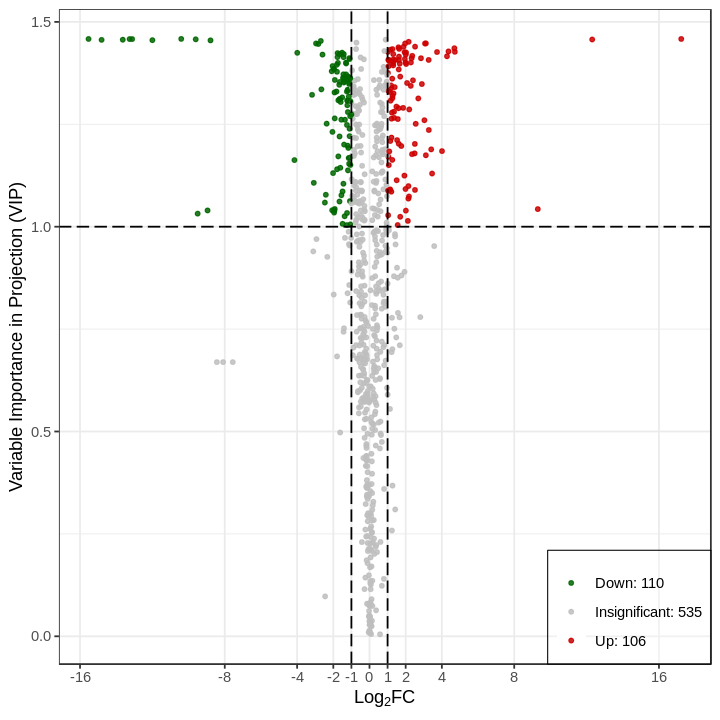

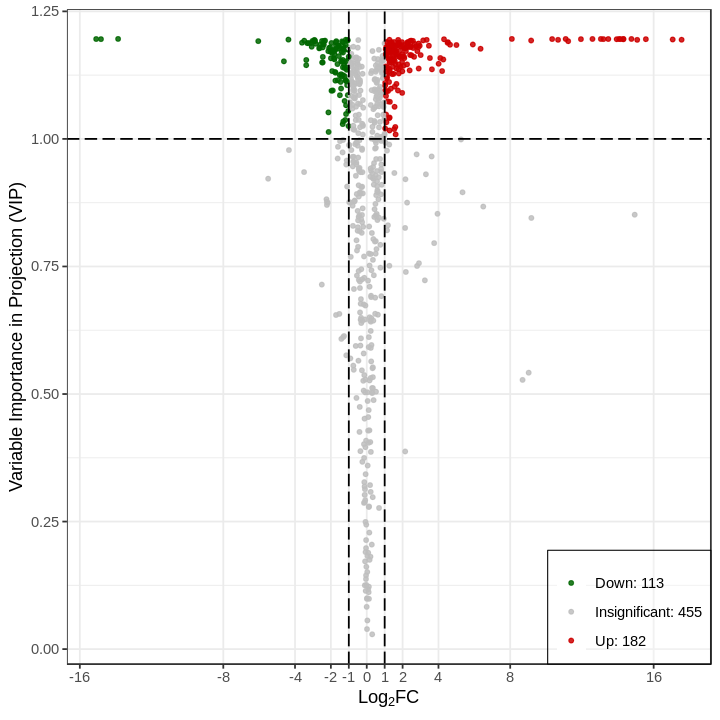


E F


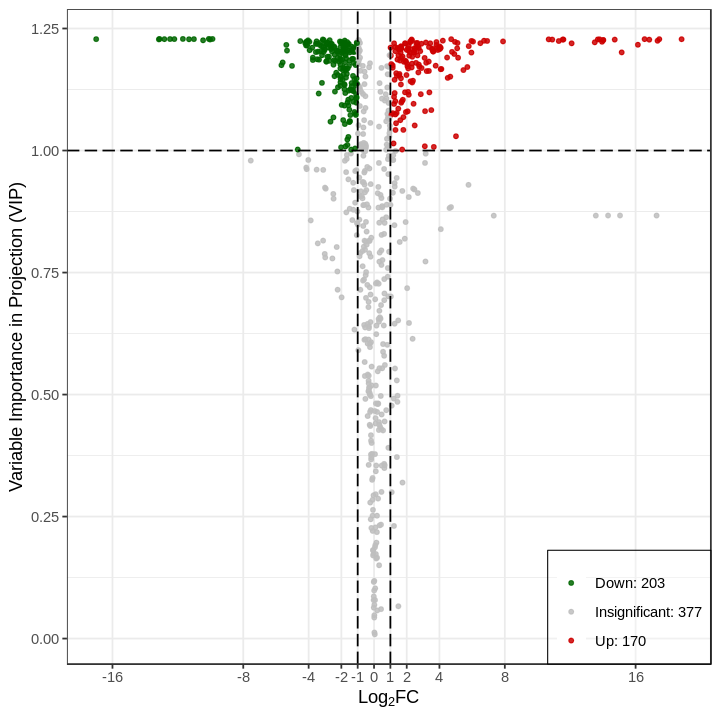

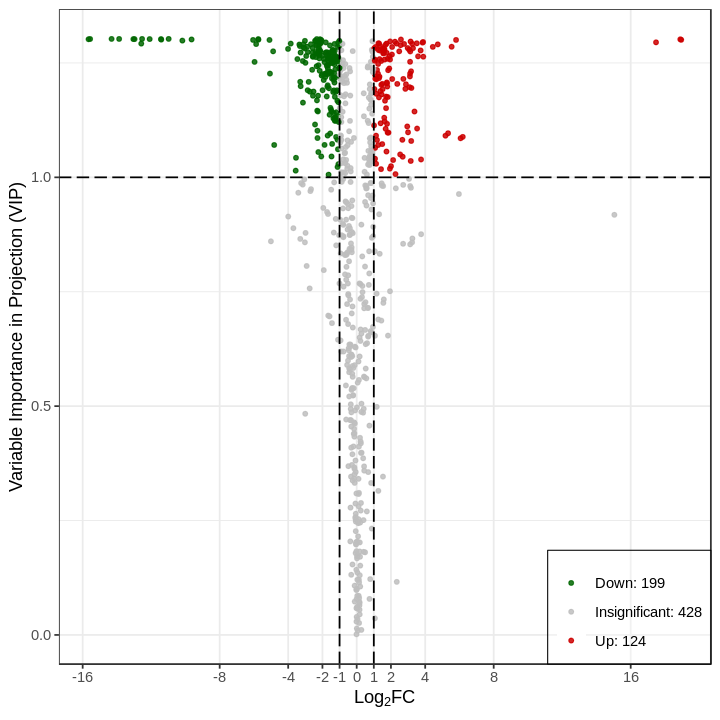


Fig. S3 Volcano plot of differential metabolites for 8M_vs_9M (A), 8M_vs_10M (B), 8M_vs_11M (C), 9M_vs_10M (D), 9M_vs_11M (E), and 10M_vs_11M (F). Each point in the figure represents a metabolite. Green points represent down-regulated metabolites, red points represent up-regulated metabolites, and gray points represent metabolites that were detected but not significantly different.


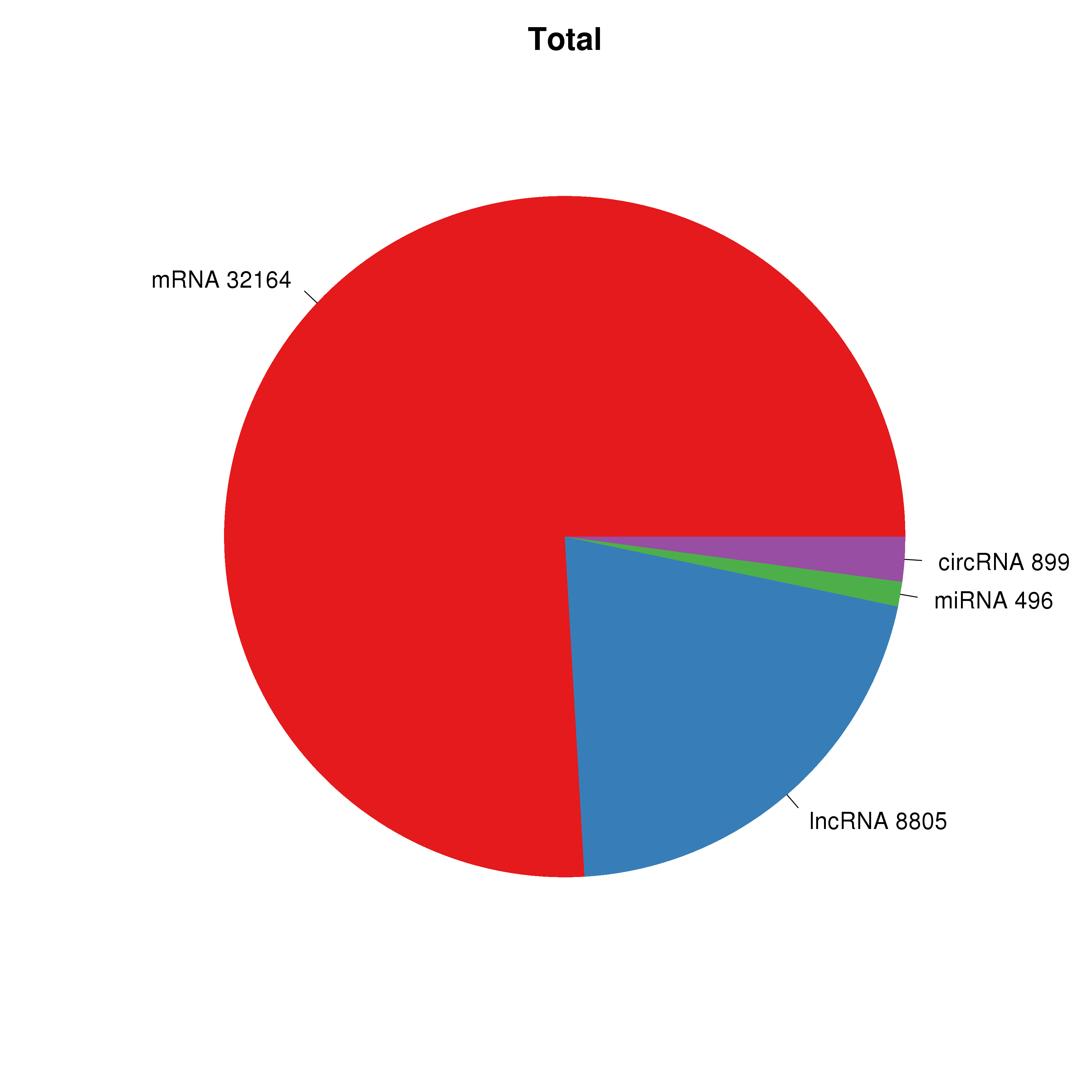

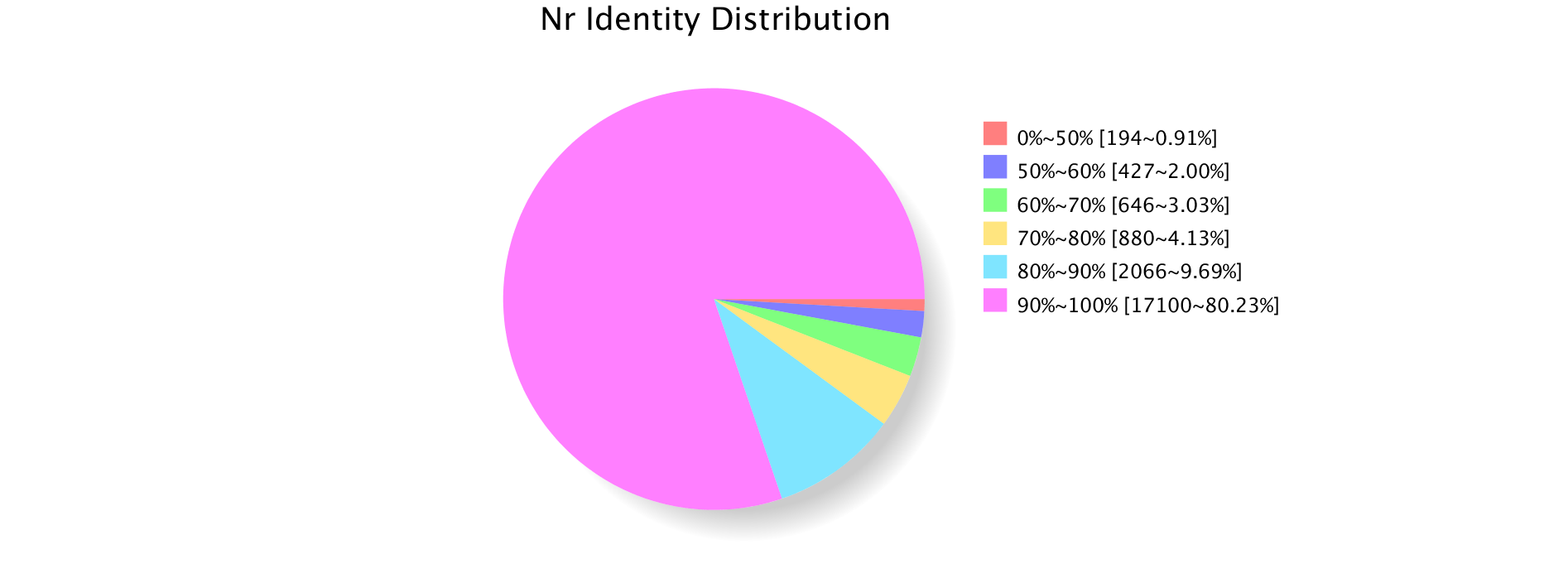

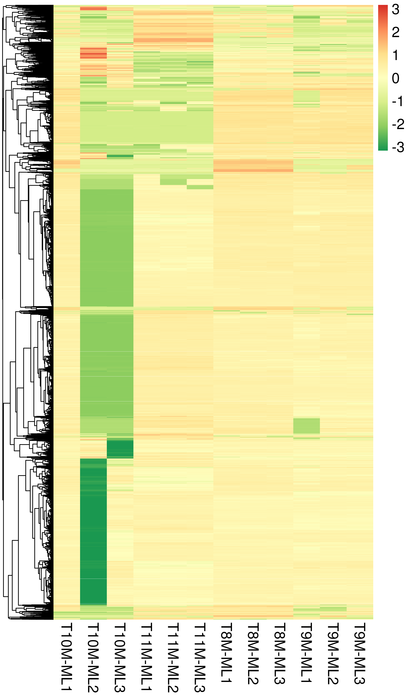
A C

B

Fig. S4 (A) A pie diagram showing the number of mRNAs, lncRNAs, miRNAs and circRNAs we identified. (B) Annotated statistics of new genes in NR database. (C) Heat map showing expression profiles of DE-mRNAs.


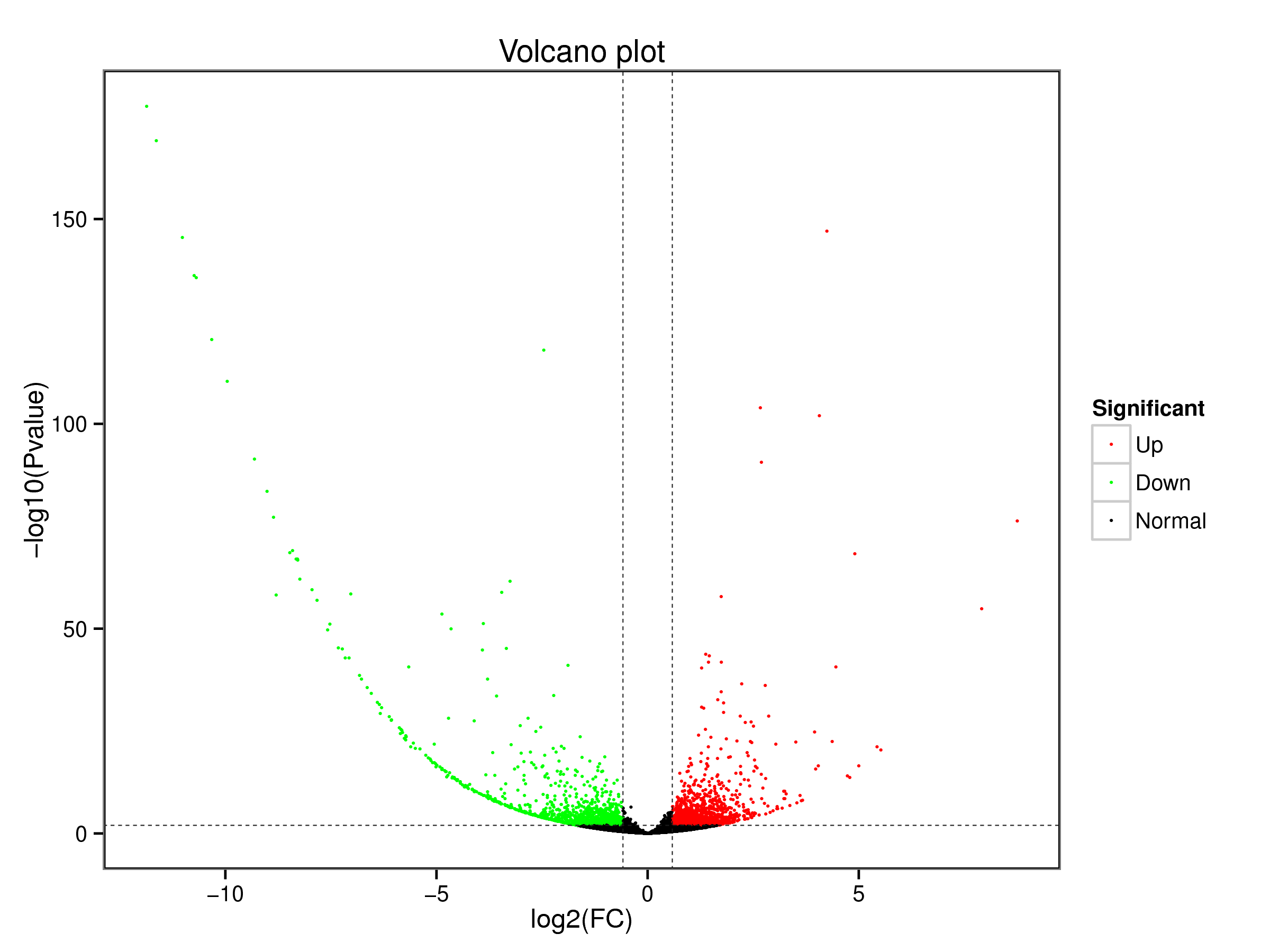
A B


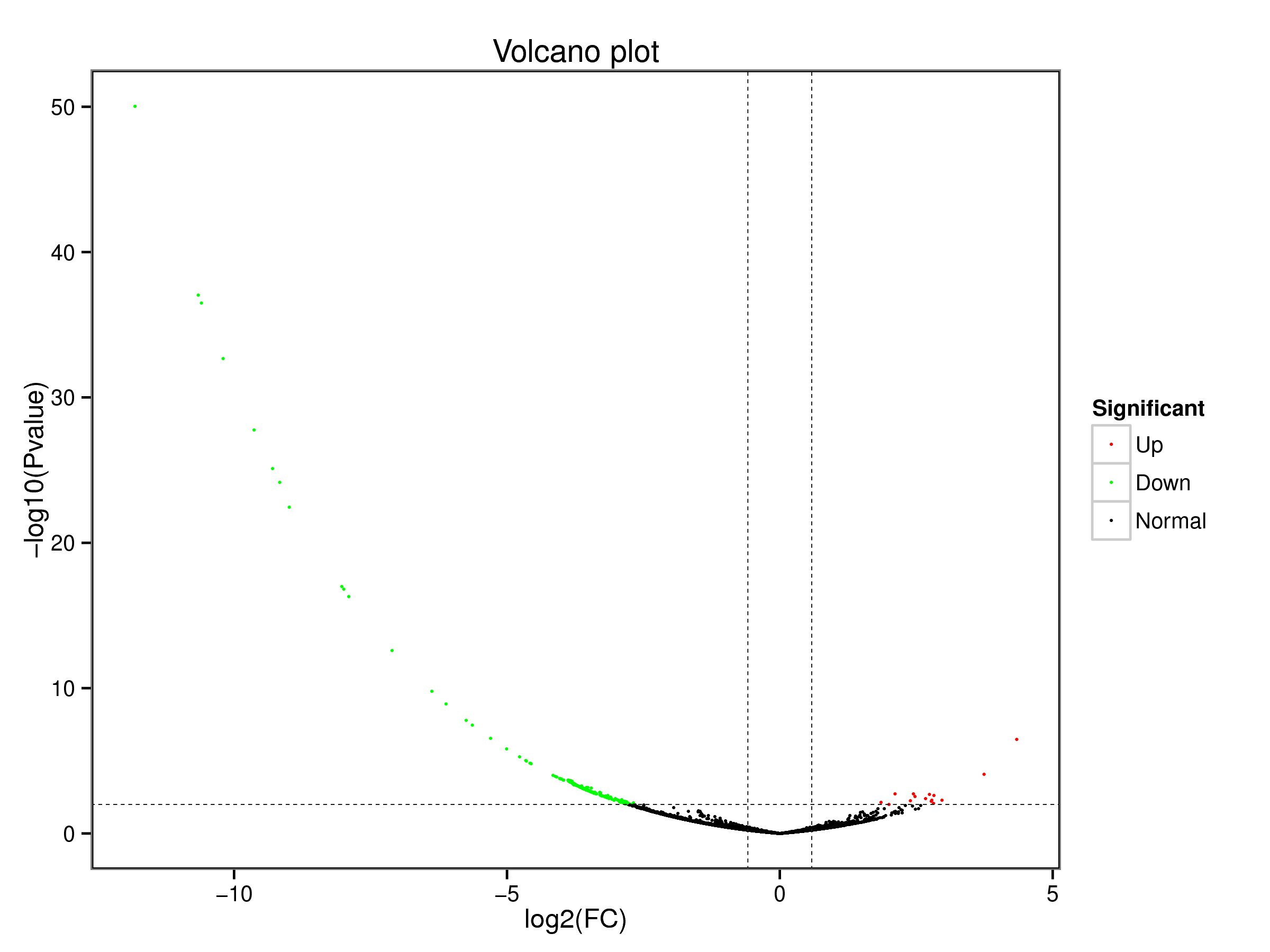

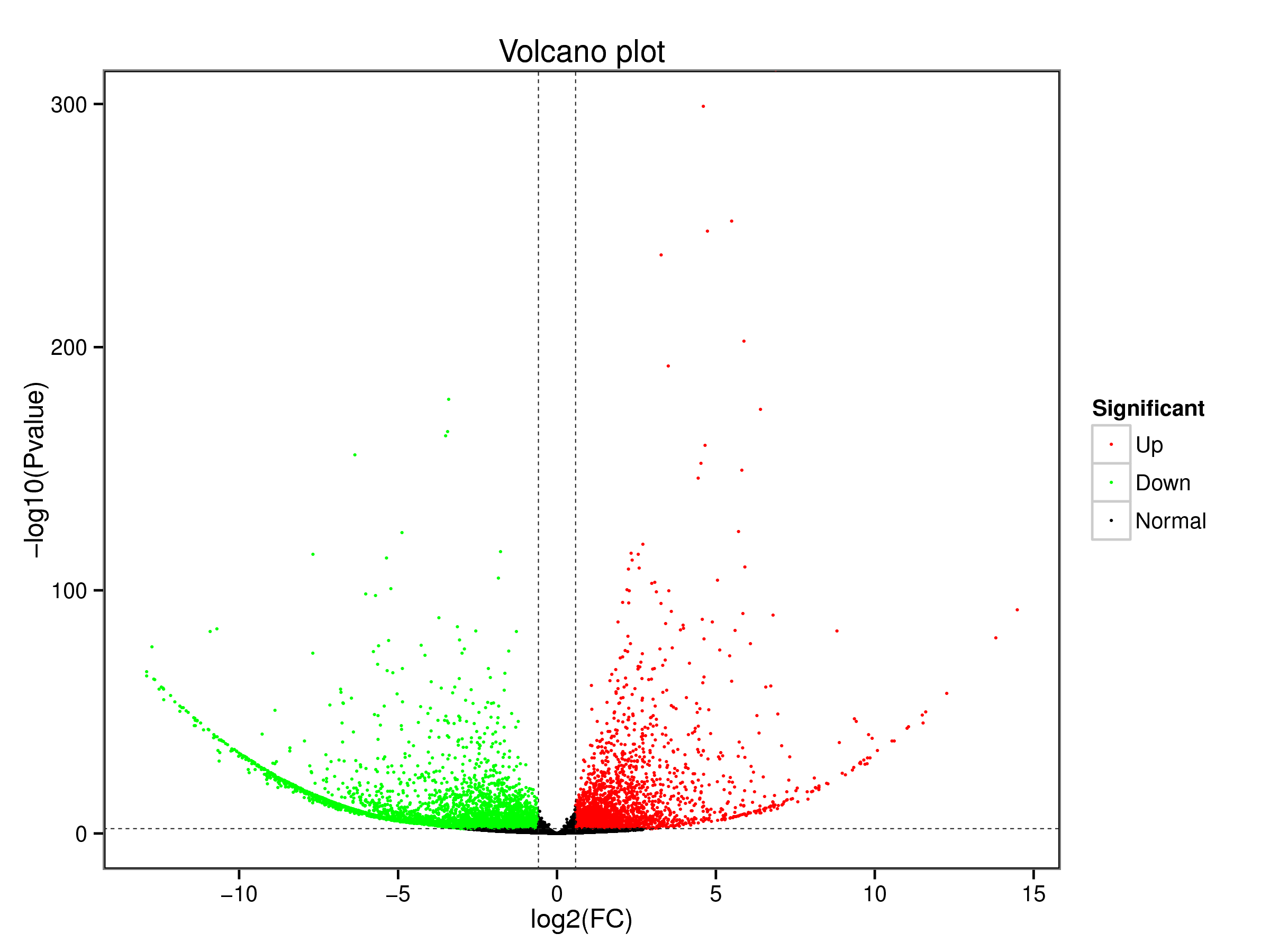

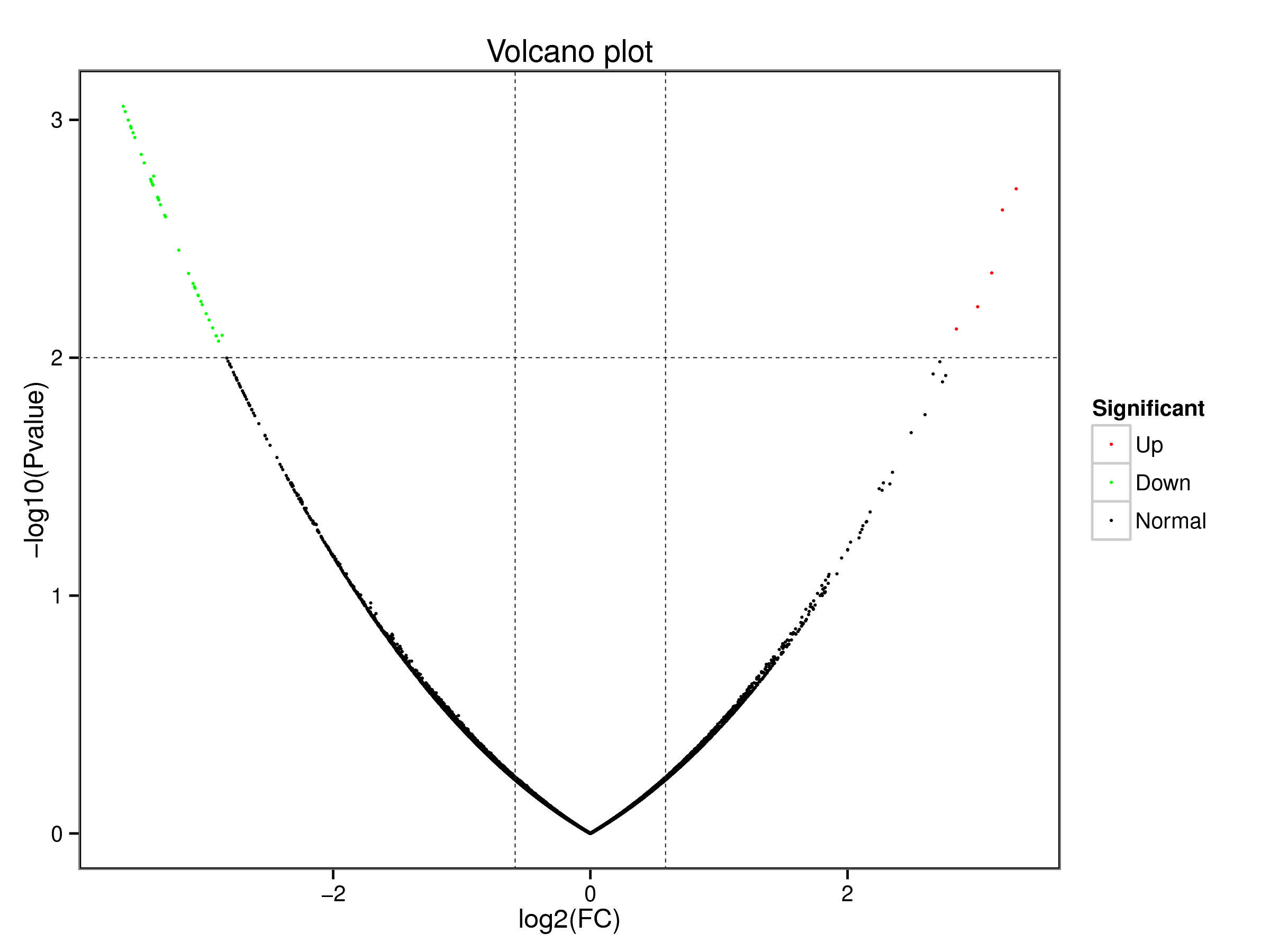
C D


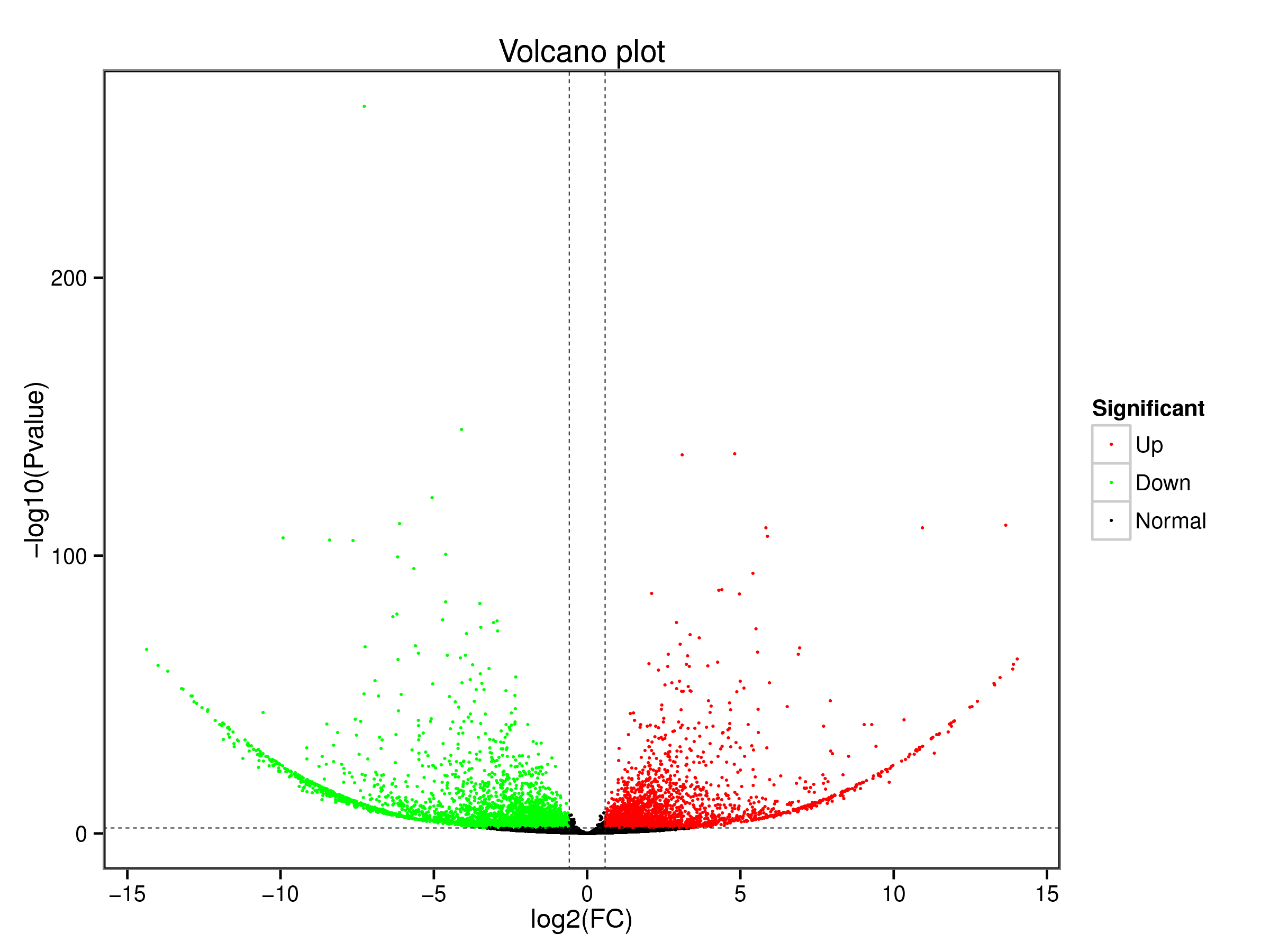
E F


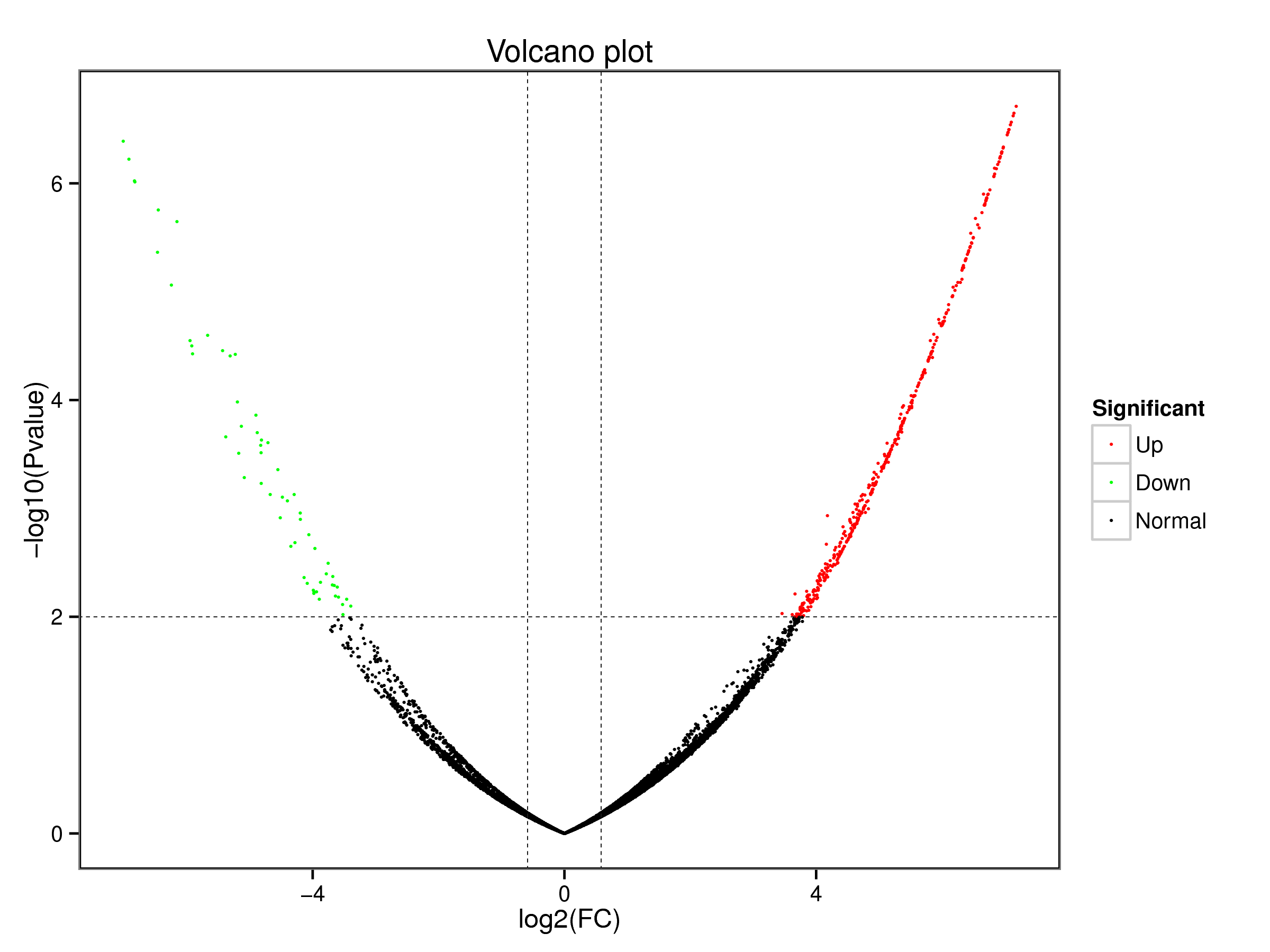


Fig. S5 Volcano plot of DEGs for 8M_vs_9M (A), 8M_vs_10M (B), 8M_vs_11M (C), 9M_vs_10M (D), 9M_vs_11M (E), and 10M_vs_11M (F). Each point in the figure represents a genes. Green points represent down-regulated genes, red points represent up-regulated genes, and gray points represent genes that were detected but not significantly different.

A B


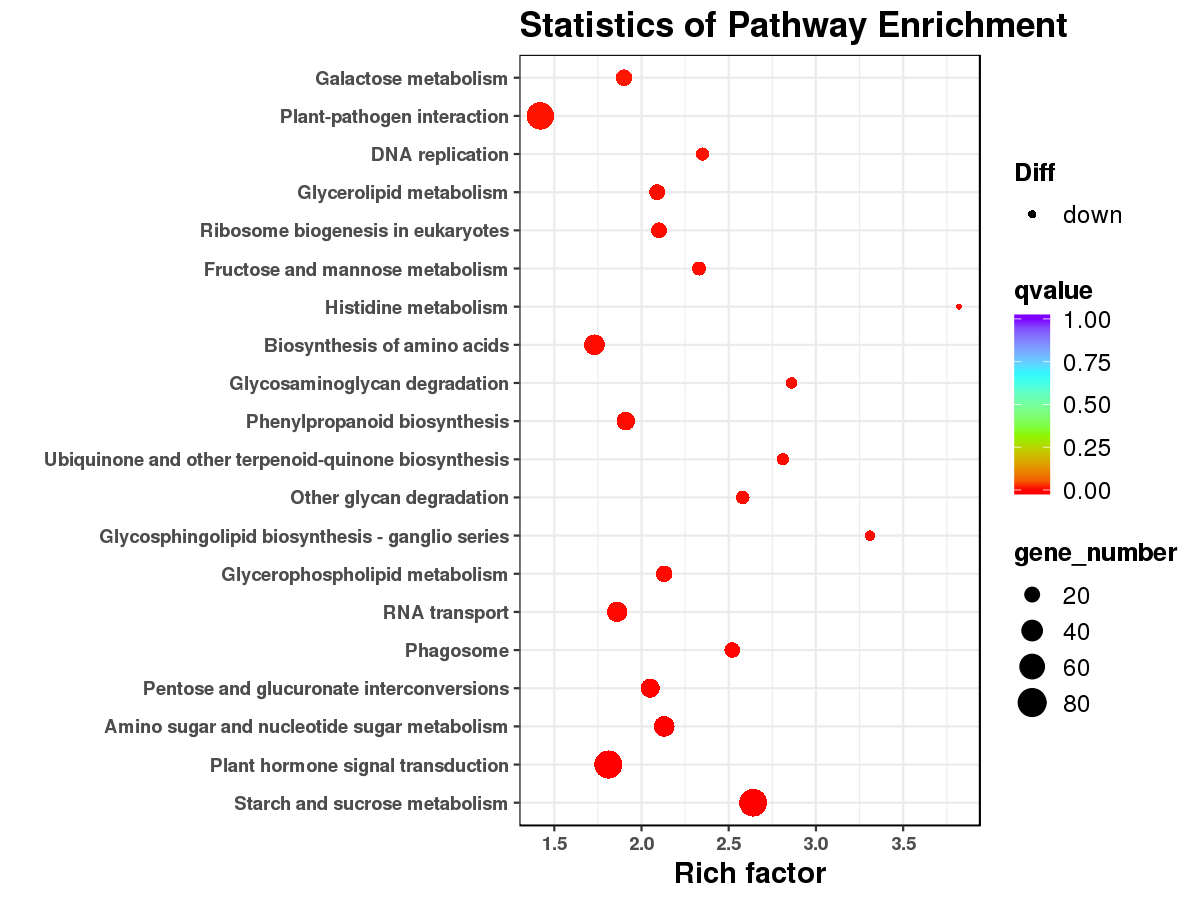

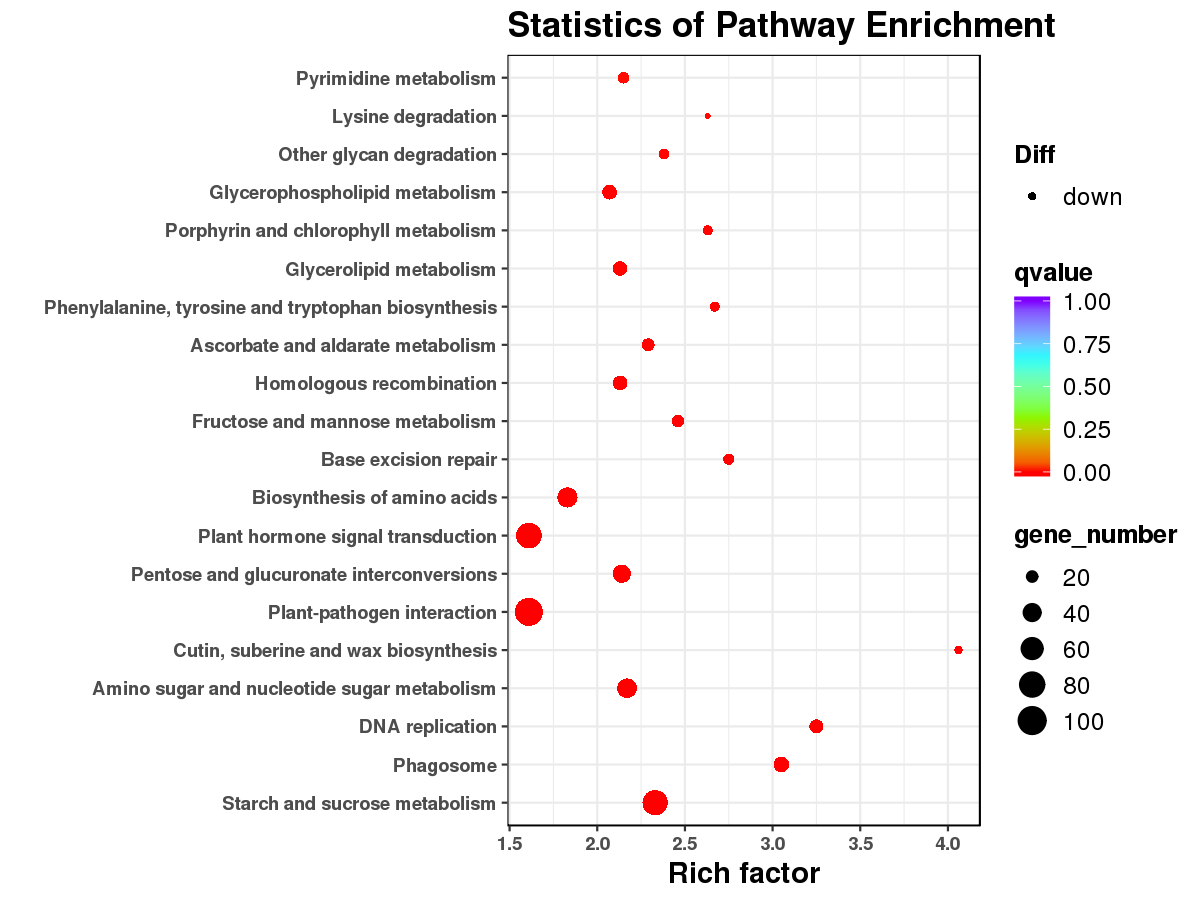


Fig. S6 KEGG enrichment analysis showed the potential functions of down-regulated genes in the 8M_vs_11M (A) and 9M_vs_11M (B) groups.

A B


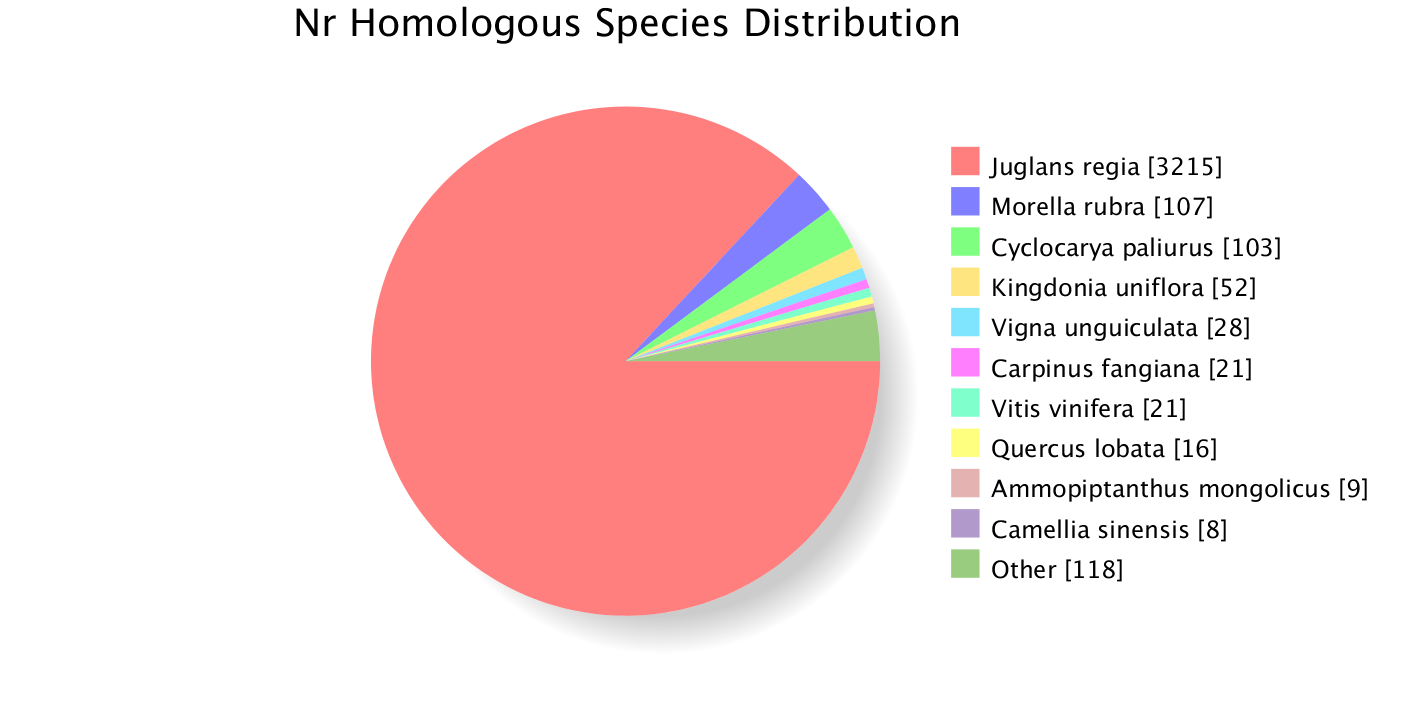

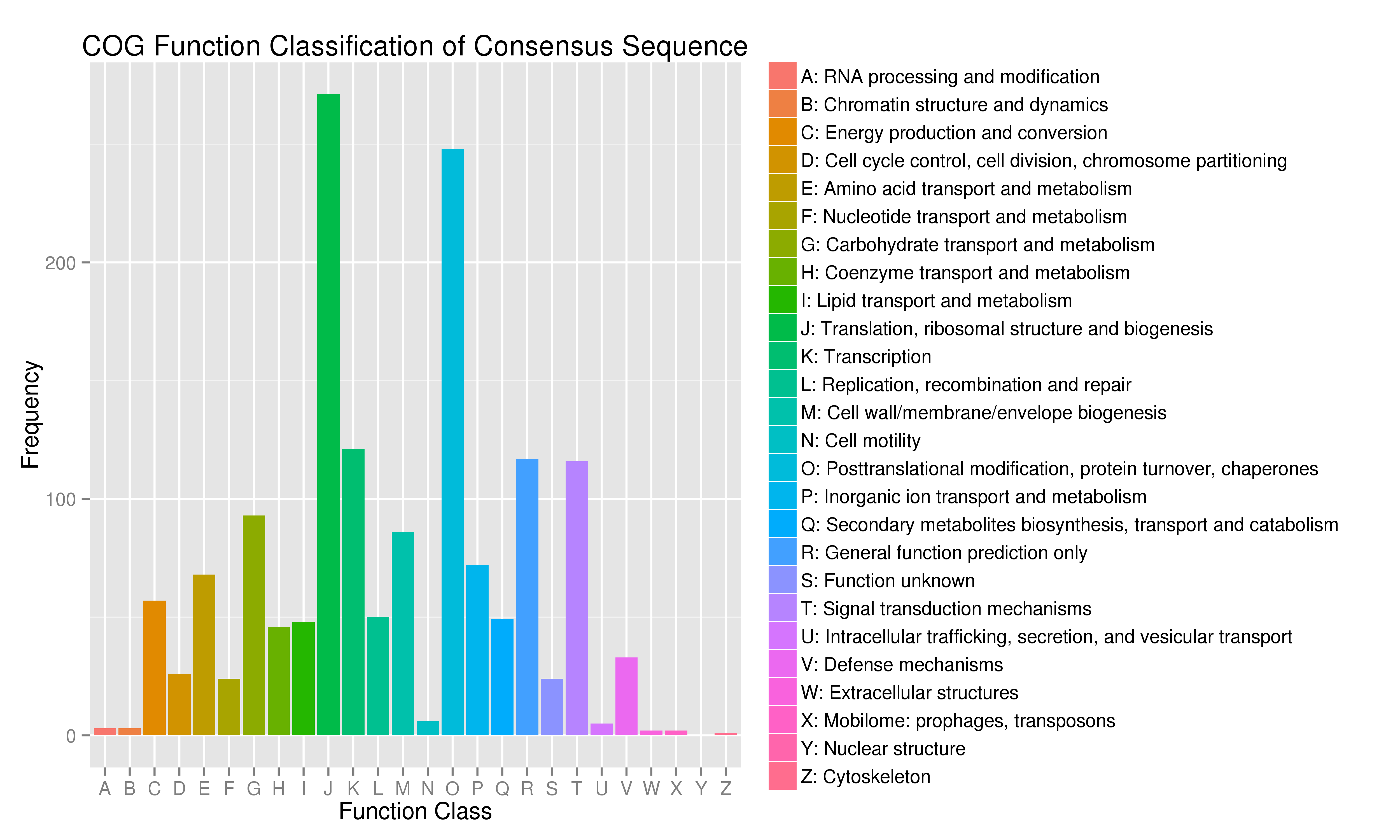


C


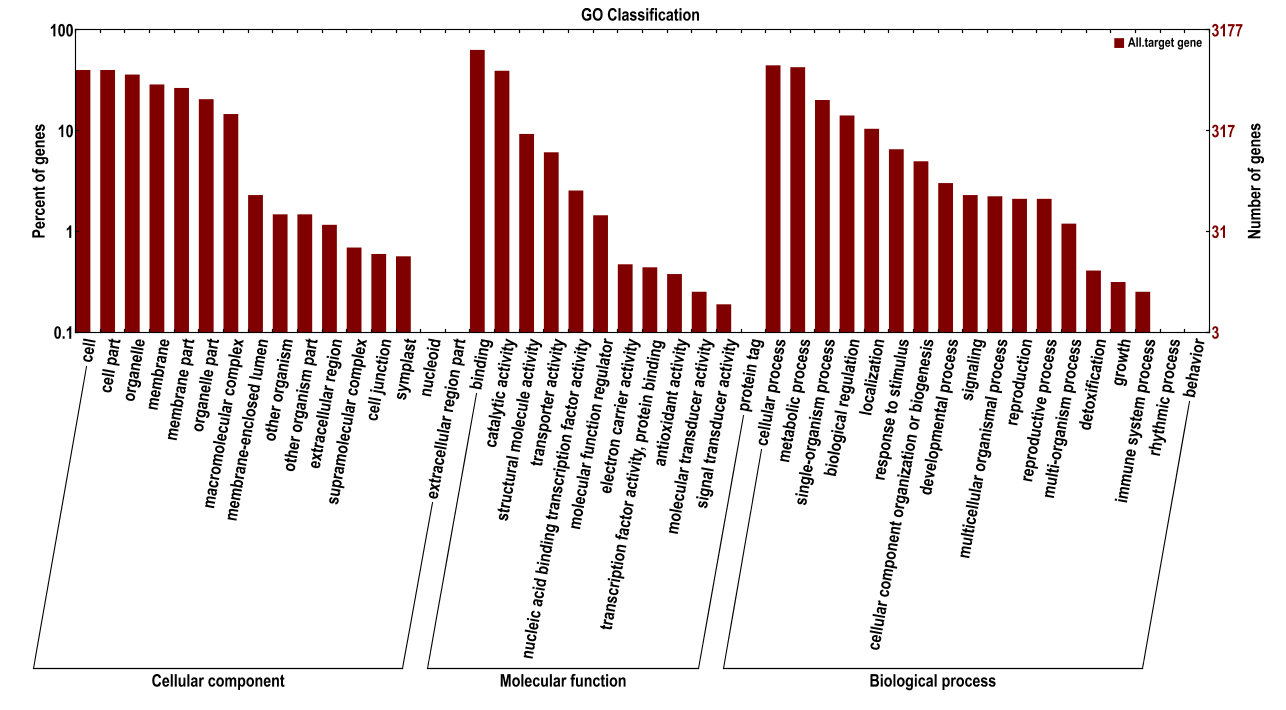


Fig. S7 Annotated statistics of miRNAs target genes in COG (A), GO (B), and NR (C) databases.

A B


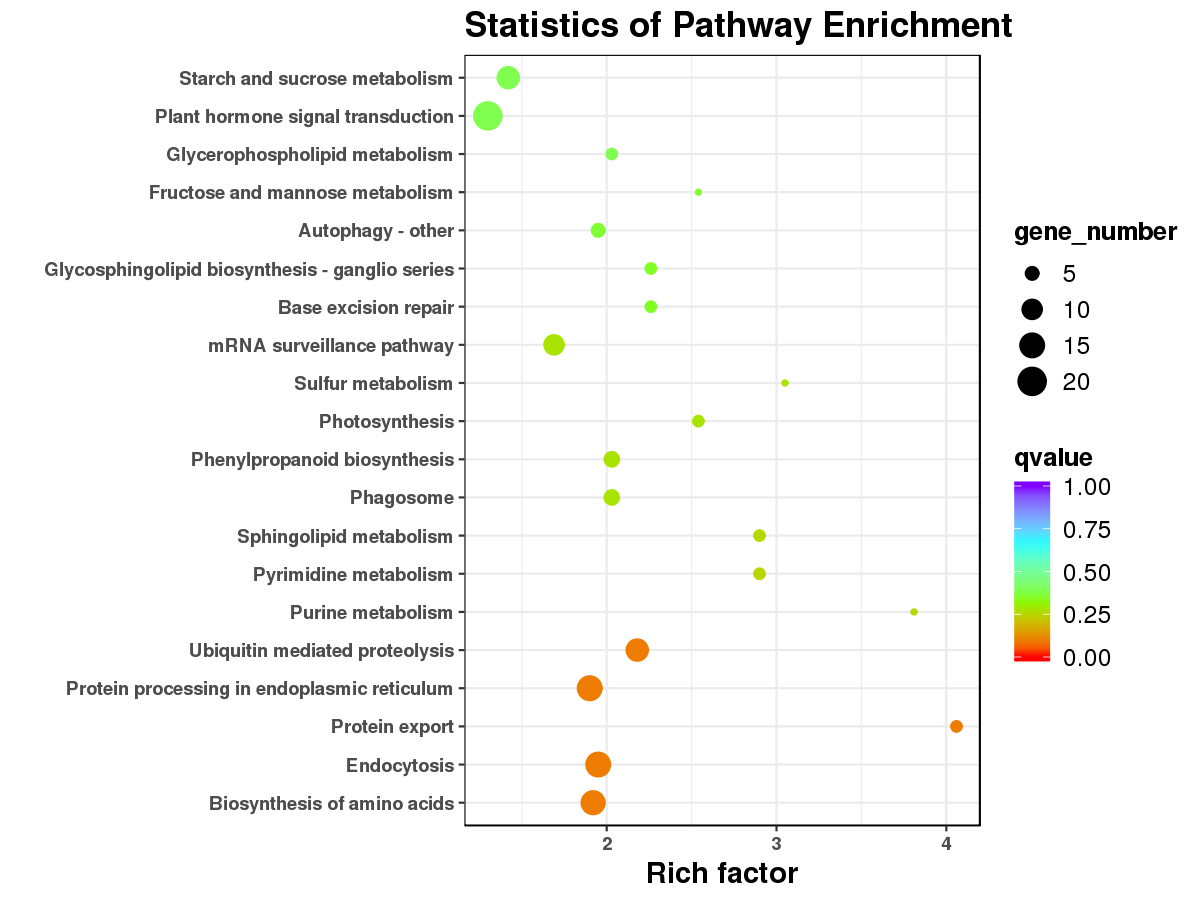

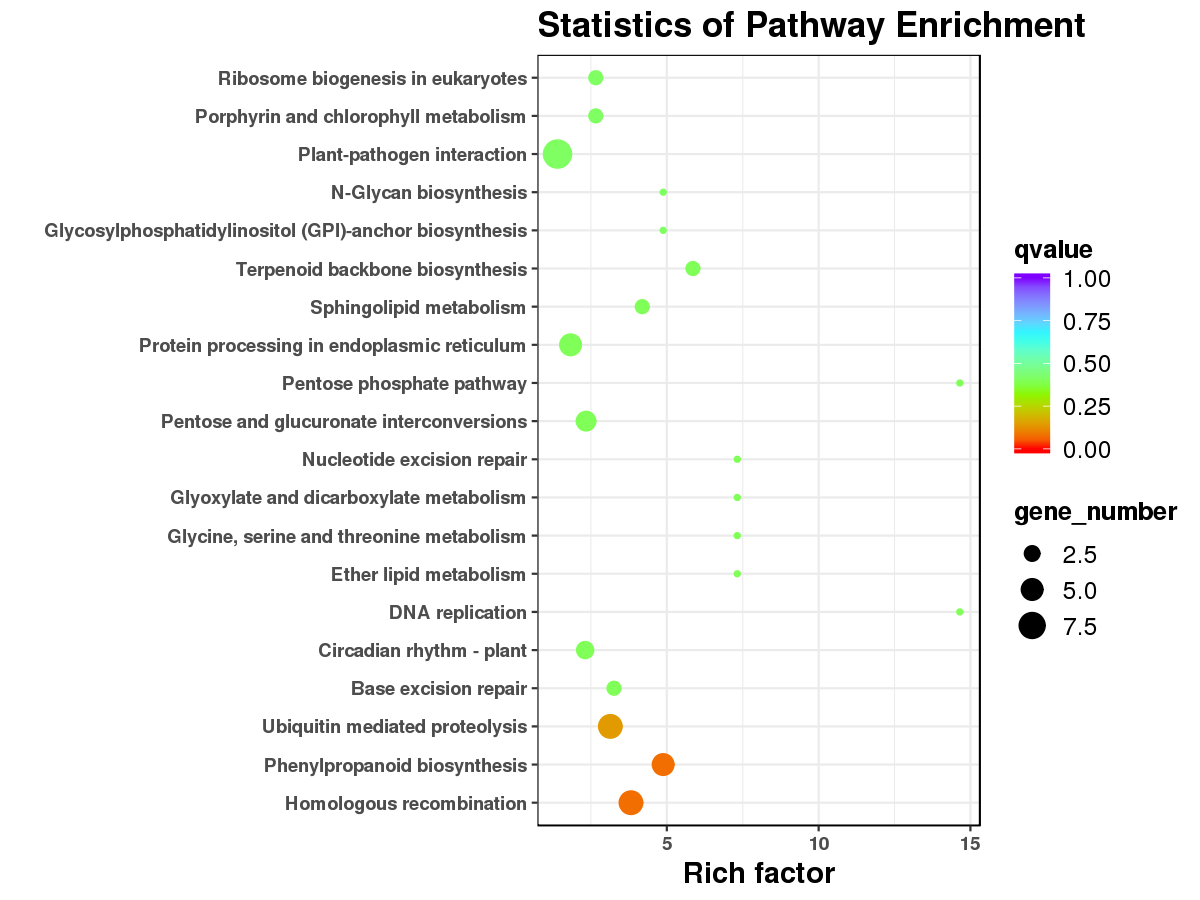

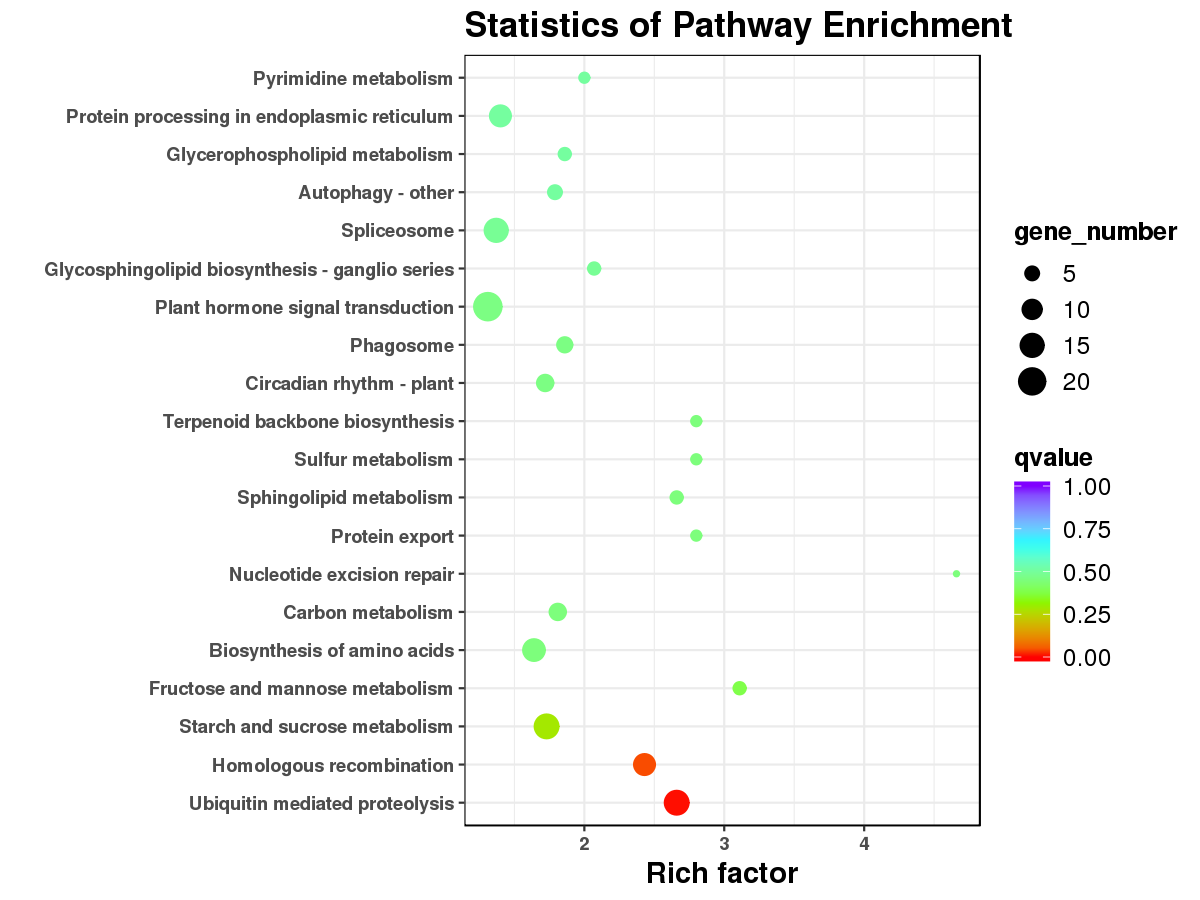
C D


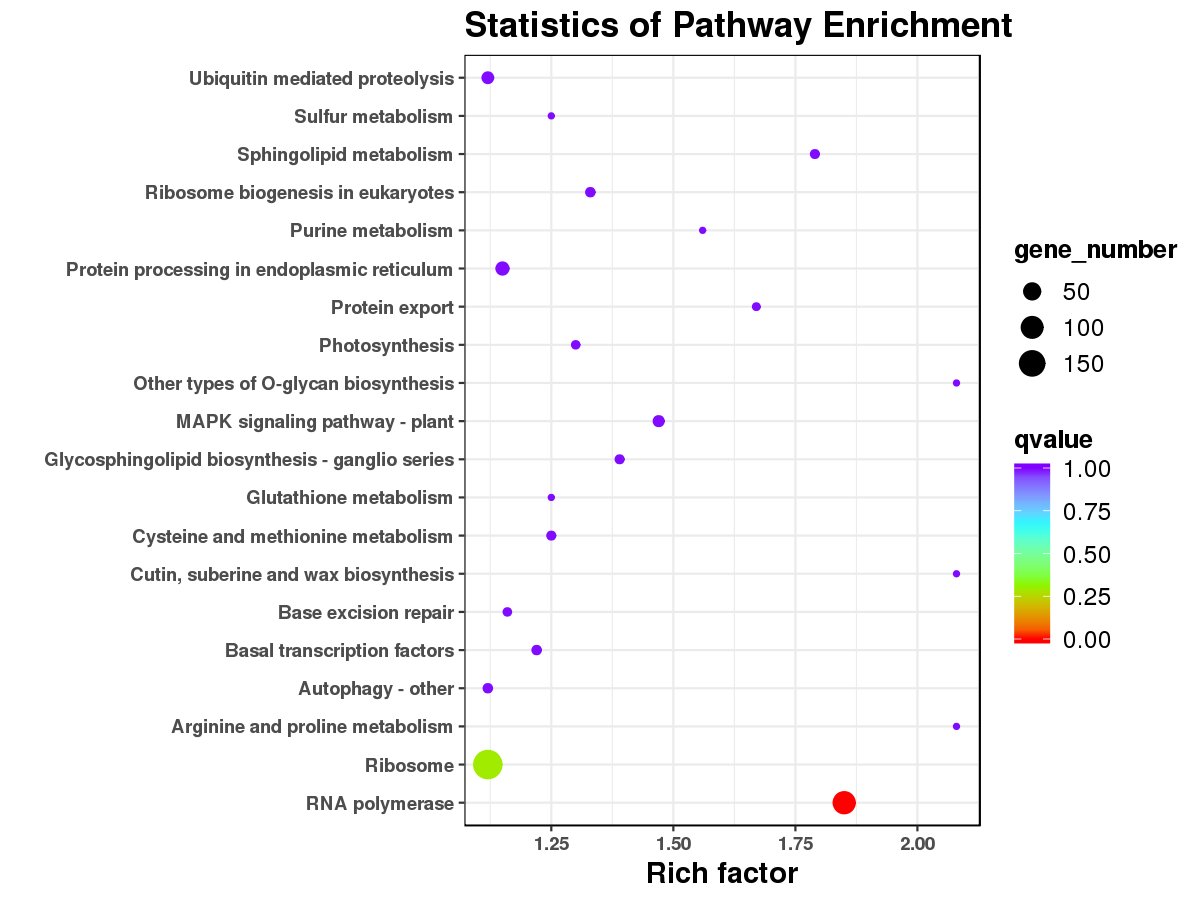
E F


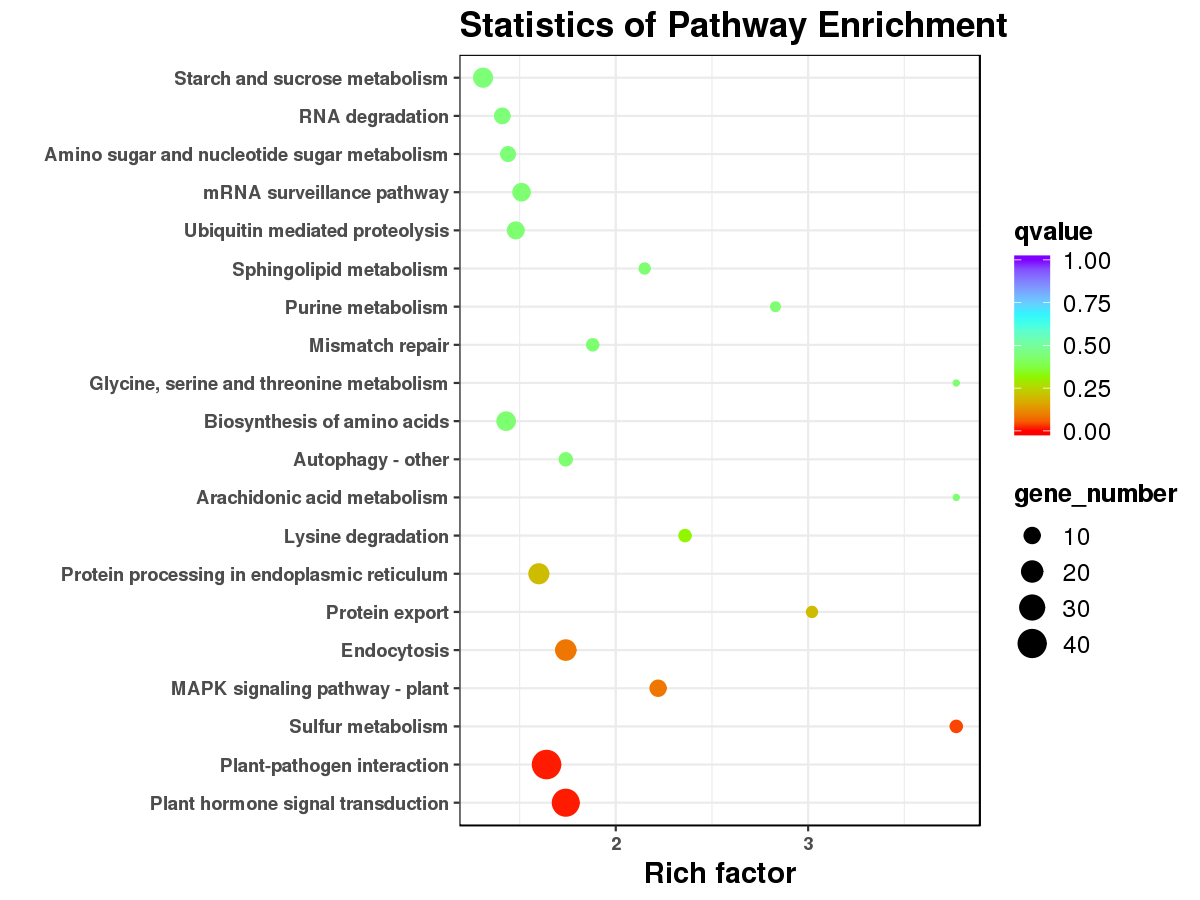

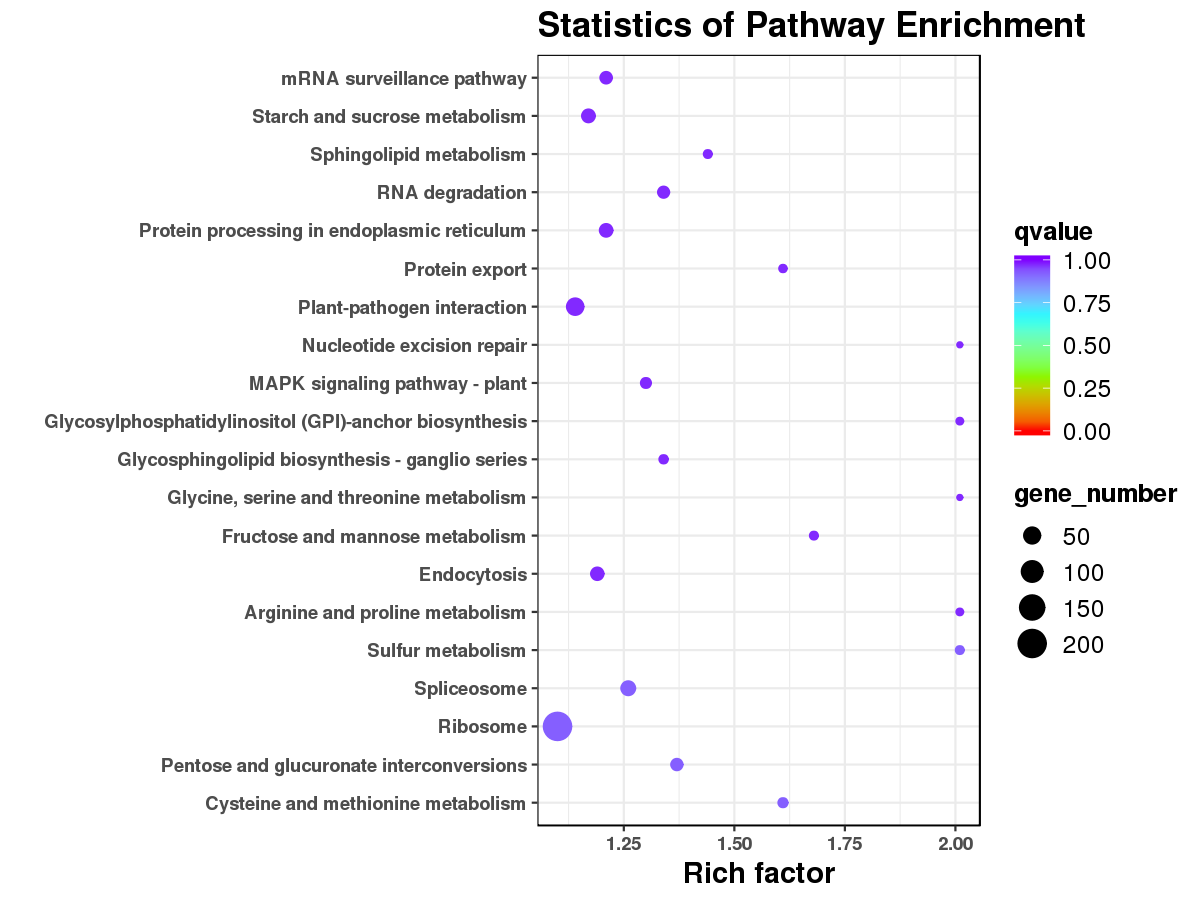
Fig. S8 KEGG enrichment analysis showed the potential functions of DE-miRNA target genes in the 8M_vs_9M (A), 8M_vs_10M (B), 8M_vs_11M (C), 9M_vs_10M (D), 9M_vs_11M (E), and 10M_vs_11M (F) groups.


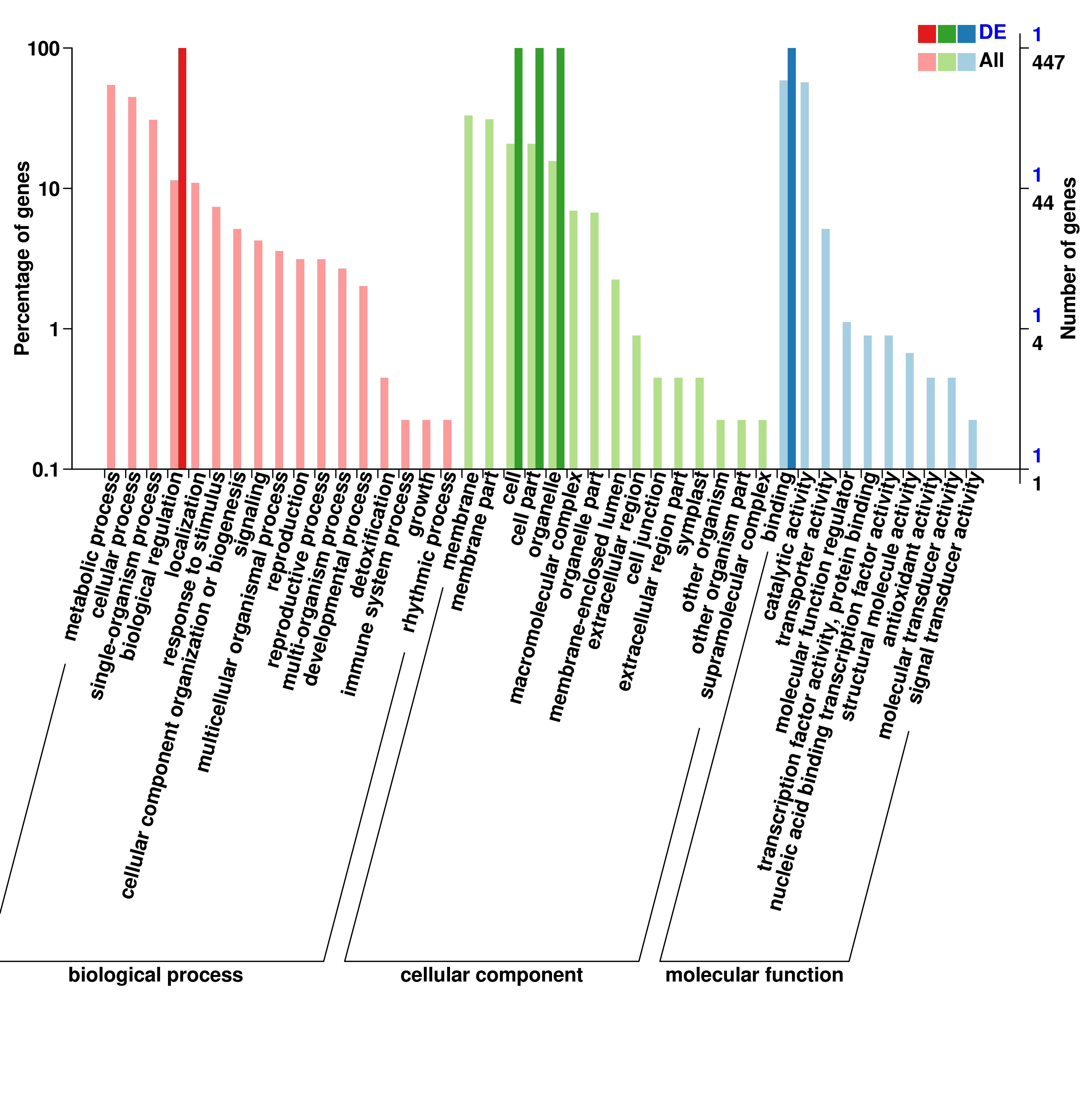
A B


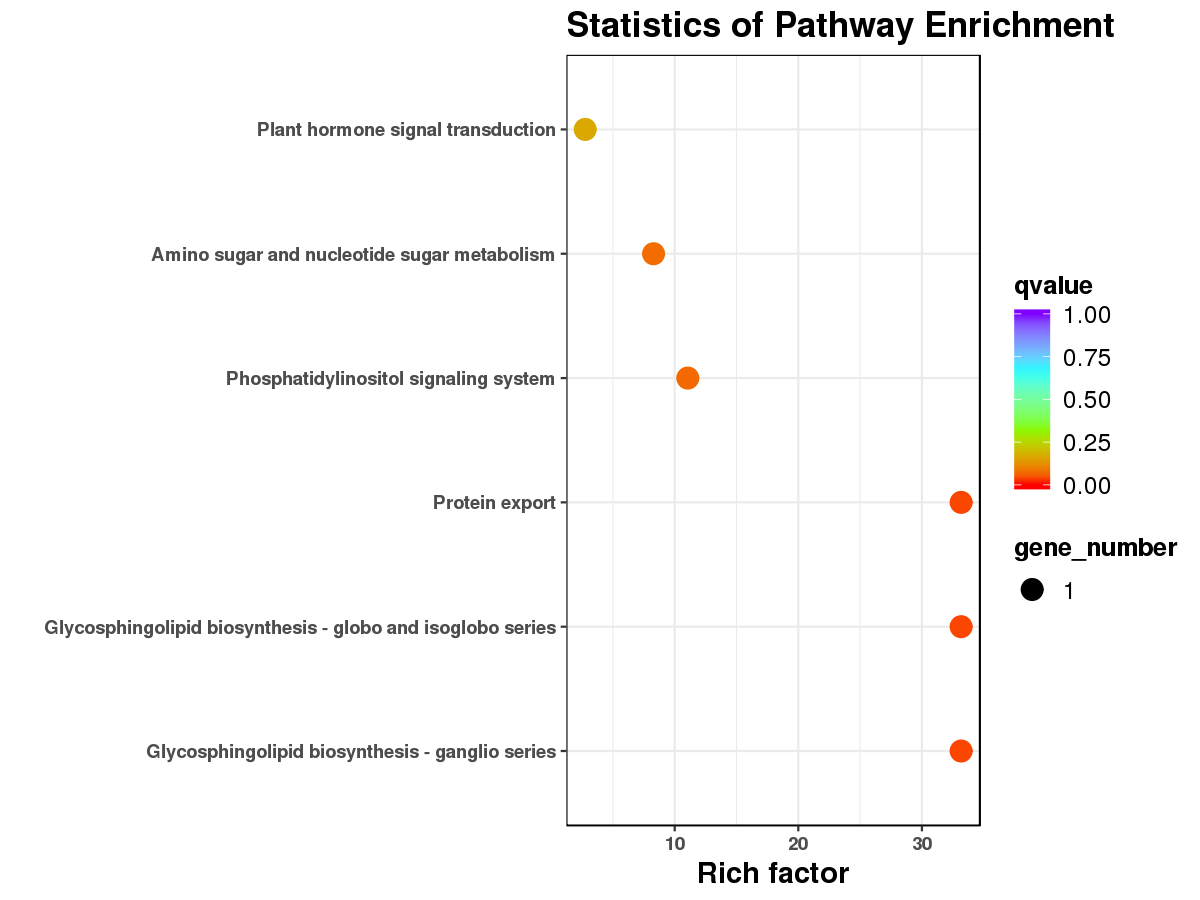
Fig. S9 Enrichment analysis showed the potential functions of DE-circRNA in the 8M_vs_11M (A) and 9M_vs_11M (B) groups.

A B


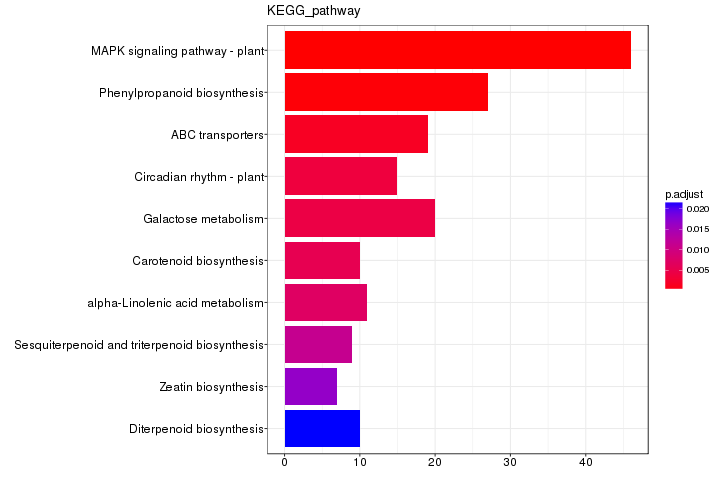

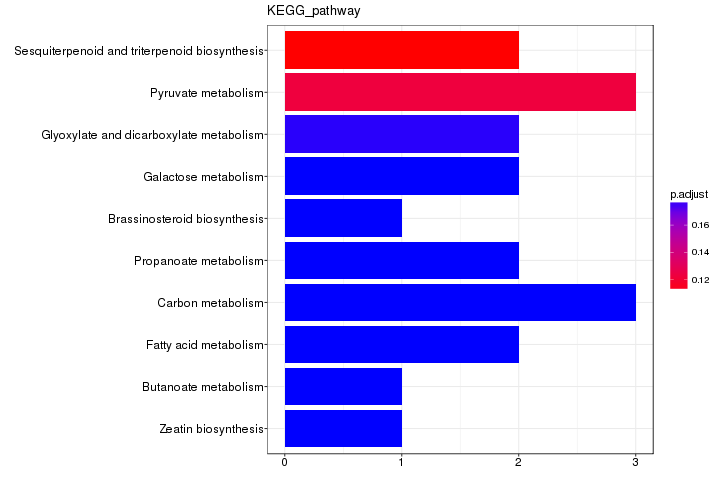
C D


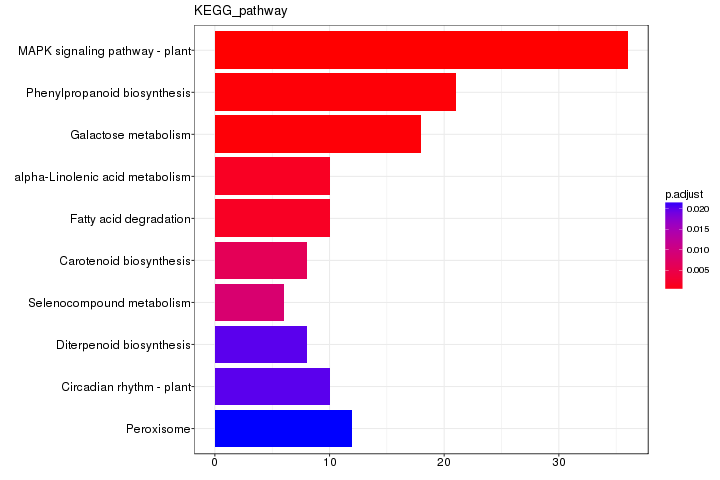

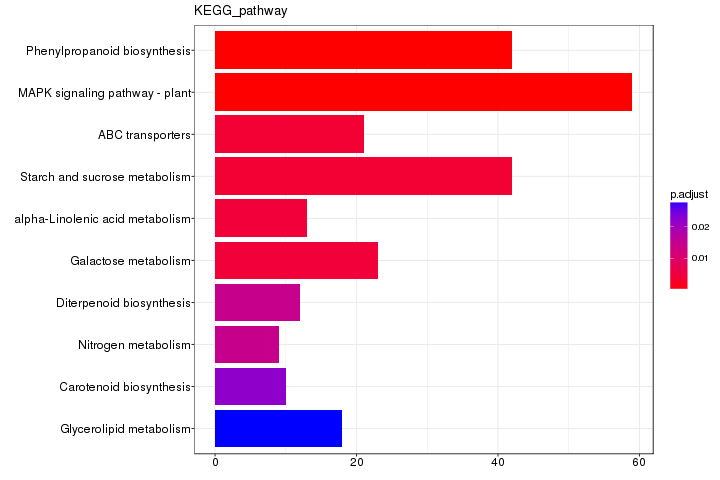
Fig. S10 Enrichment analysis showed the potential functions of the relationship pairs from DE-ceRNAs in the 8M_vs_9M (A), 8M_vs_11M (B), 9M_vs_11M (C), and 10M_vs_11M (D) groups.


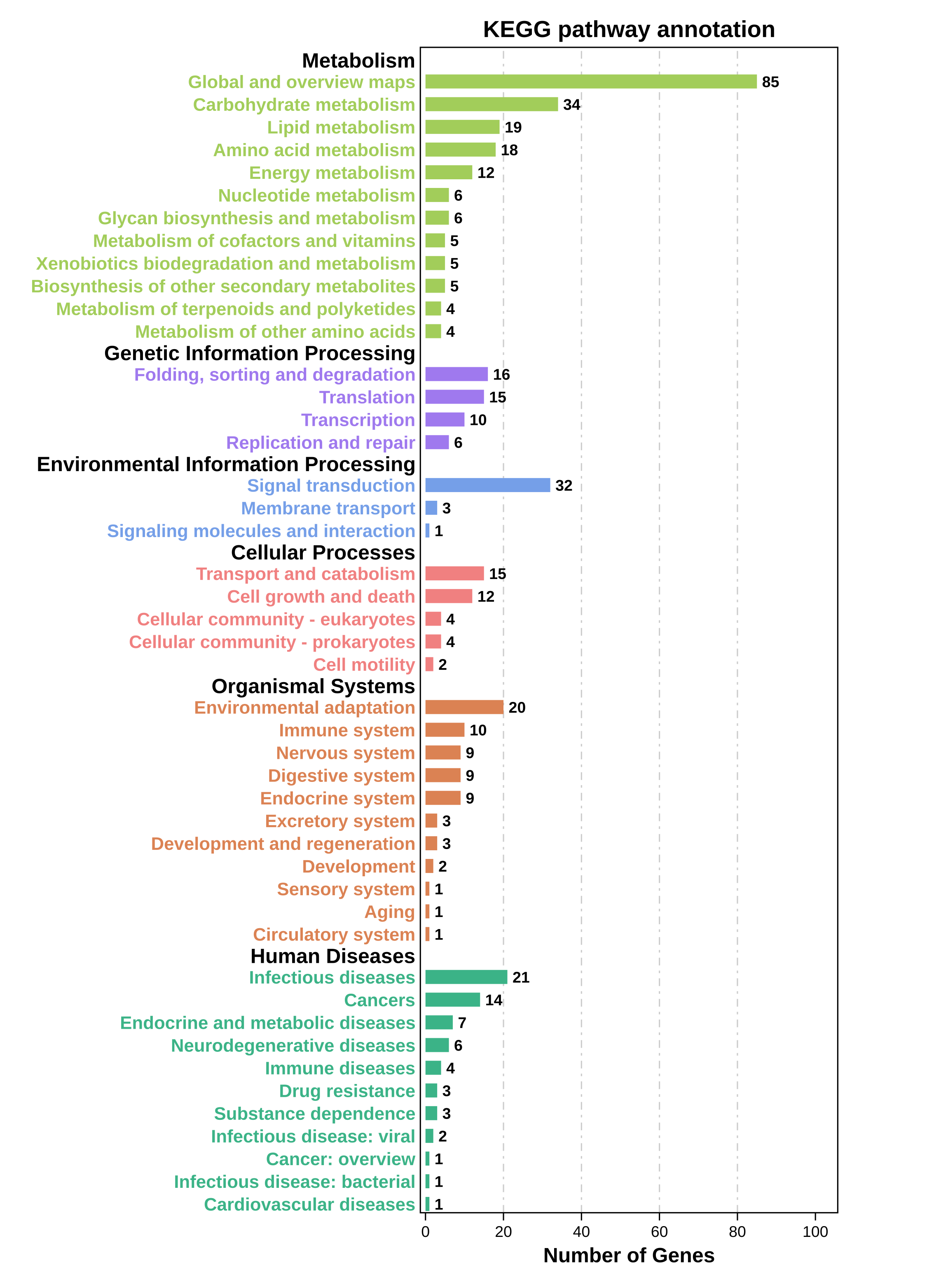


Fig. S11 KEGG enrichment analysis showed the potential functions of genes related to polysaccharides in the green module.

A B


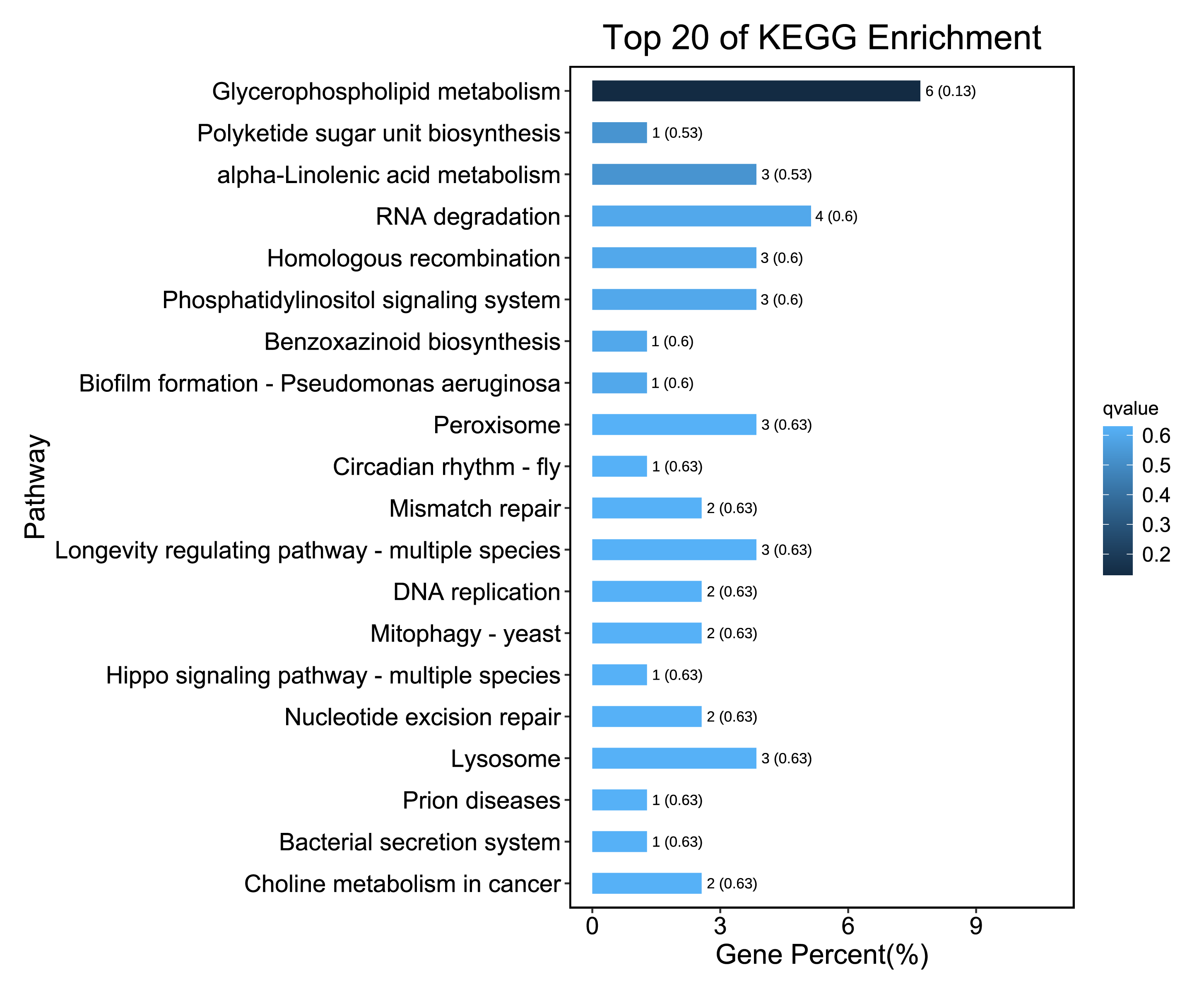

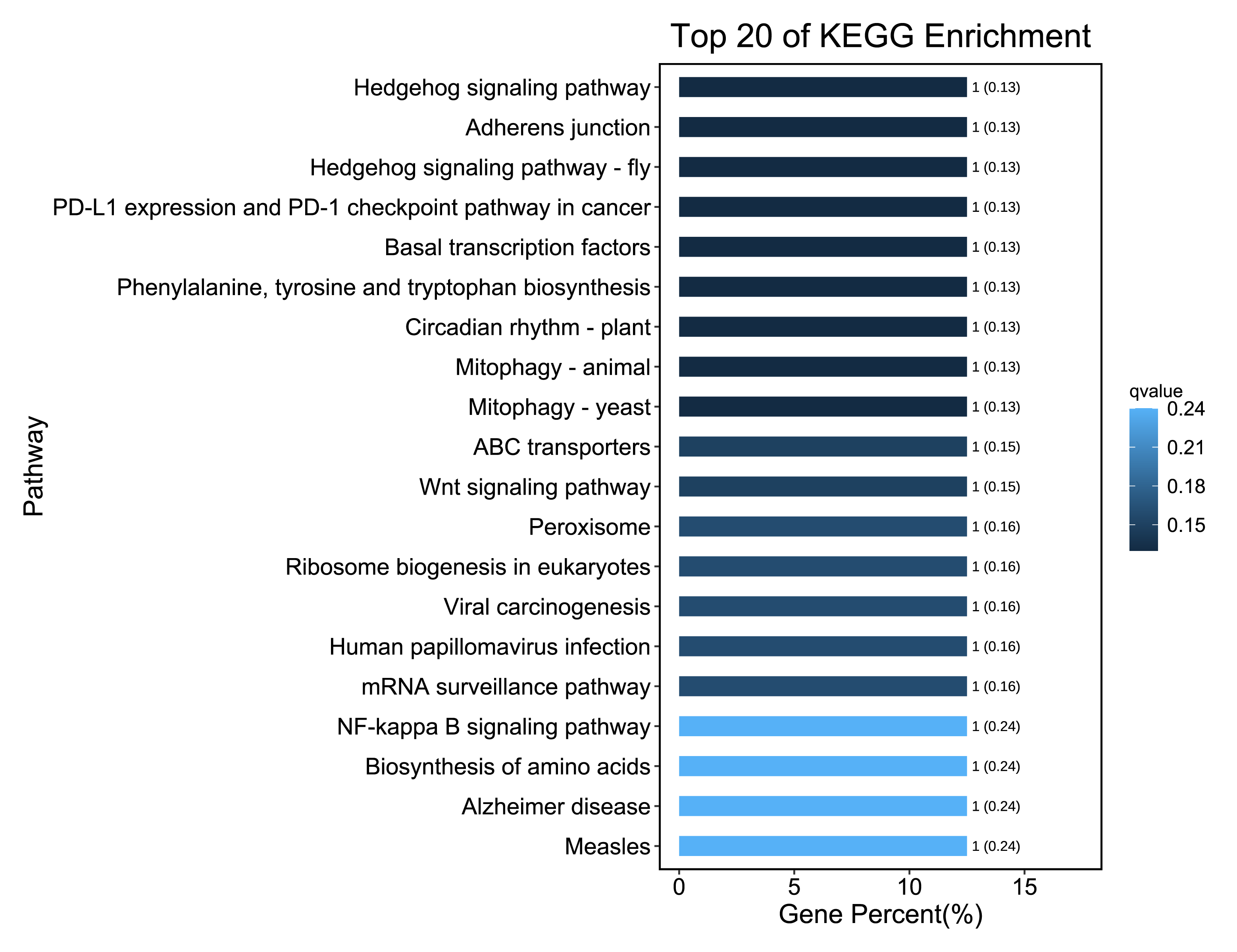


Fig. S12 KEGG enrichment analysis showed the potential functions of genes related to polysaccharides in the greenyellow (A) and darkred modules (B).


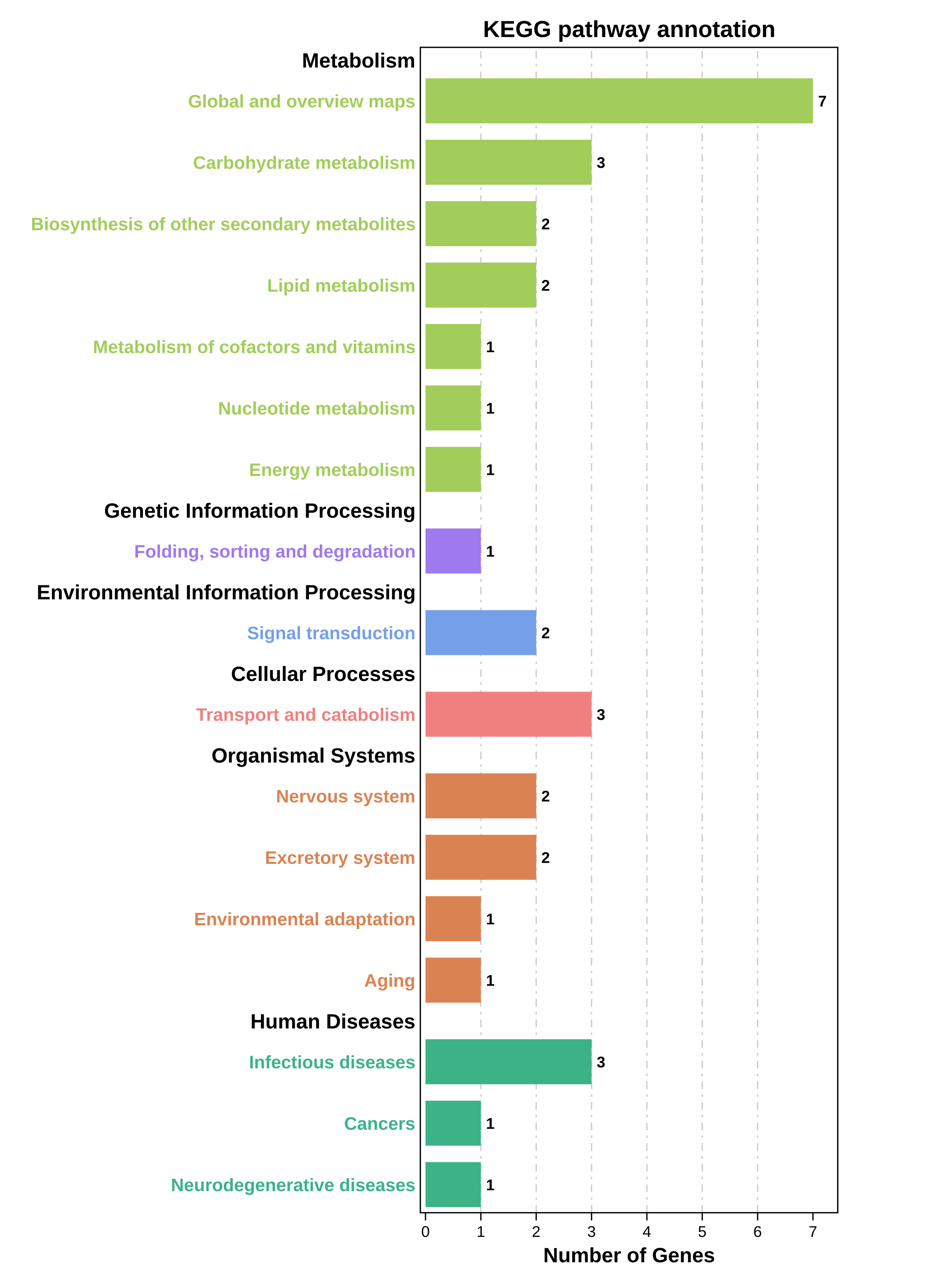


Fig. S13 KEGG enrichment analysis showed the potential functions of genes related to flavonoids in the lightyellow module.


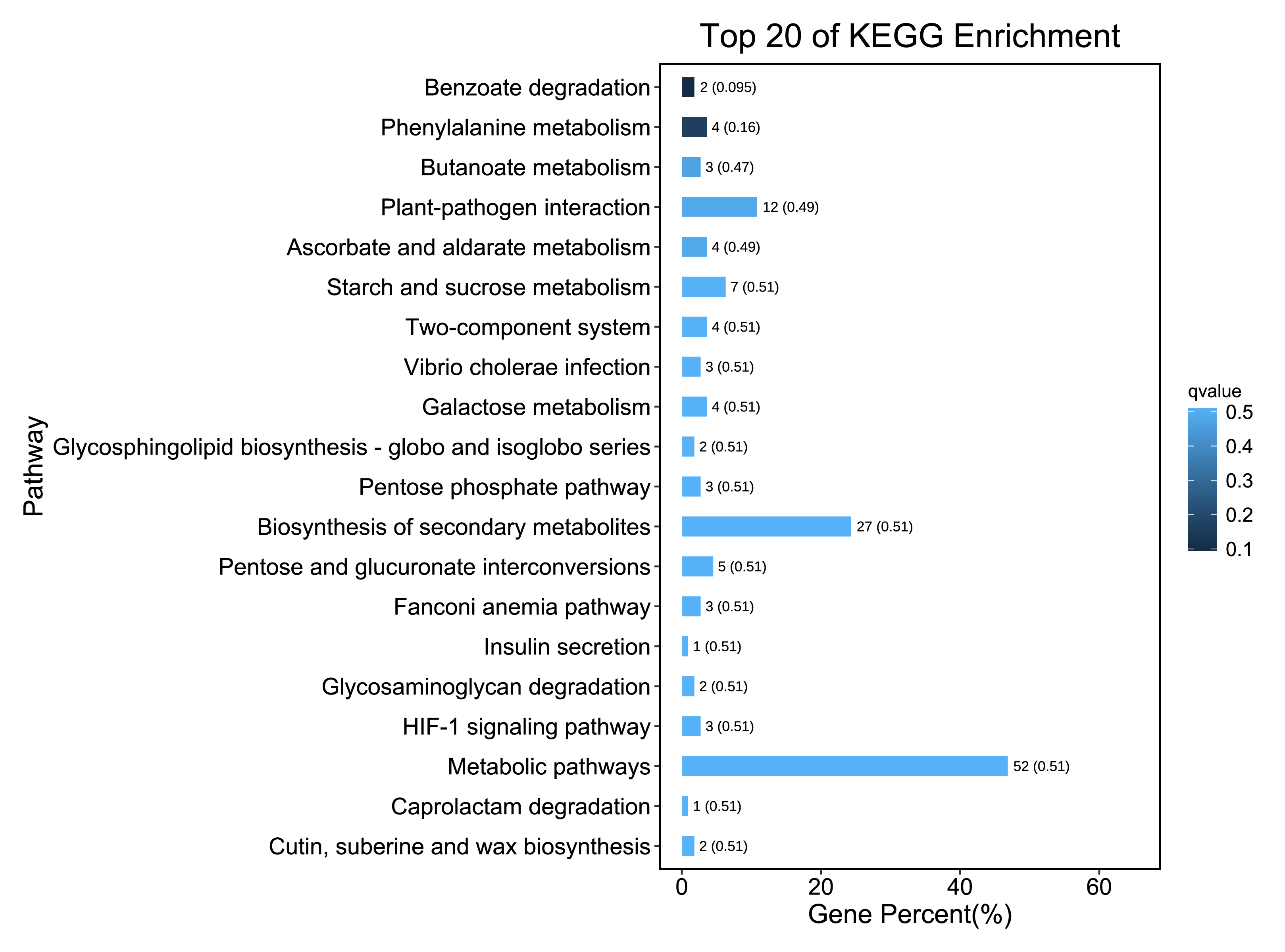


Fig. S14 KEGG enrichment analysis showed the potential functions of genes related to triterpenoids in the black module.

A B C

D E

Fig. S15 Real-time quantitative RT-qPCR confirmation of *CpSUS*, *CpWRKY1*, *CpFK*, *CpHCT*, and *Cp4CL*. Relative gene expressions were analyzed using the 2^−ΔΔCt^ method. Experiments were performed in triplicate. Error bars indicate standard deviation.
