# Supplementary material for: Metabolome and Whole-Transcriptome Analyses Reveal the Molecular Mechanisms Underlying Hypoglycemic Nutrient Metabolites Biosynthesis in Cyclocarya paliurus Leaves During Different Harvest Stages
Source: Front Nutr. 2022 Feb 28;9:851569. doi: 10.3389/fnut.2022.851569 (PMC8919051; doi:10.3389/fnut.2022.851569)
Supplement: Supplementary file 2 [file Data_Sheet_2.doc]

**Supplementary Tables**

Table S1 Primers for genes validated by quantitative real-time PCR (qRT-PCR).

| Genes | Sequences 5’-3’ |
| --- | --- |
| GDAPH-F | CCGTTCATCACCGTTGACTACA |
| GDAPH-R | AACAGTCACAGGCTTTTCACCAA |
| CpFK-F | TCTAAACCTAAATTTAGGAATCTATAAAAT |
| CpFK-R | AGTGAAGAAATGATCGATGGATC |
| CpSUS-F | ATCCAATTCAGTCTCCCATTG |
| CpSUS-R | TCAGGTATCTTTTTCTTTCTCCTG |
| CpHCT-F | CCGGCAGAAATATACGCAACA |
| CpHCT-R | AAAATGATGAAGTTTTTGCAGAC |
| Cp4CL-F | ACAAAGACCACAAACCGAGC |
| Cp4CL-R | TCCATCGGTGTTGGCTTTA |
| CpWRKY-F | GGCAGGTACAATCTTATCAAGCT |
| CpWRKY-R | CTTTCGGGTTTGGAGGGTAA |

Table S2 Data for total flavonoids content determination.

| Month | OD_420_ | Rutin (mg/mL) | Total flavonoids (mg/g) |
| --- | --- | --- | --- |
| 8M | 0.1097 | 0.1704 | 5.6784 |
|  | 0.0819 | 0.0972 | 3.2392 |
|  | 0.0934 | 0.1274 | 4.2482 |
| 9M | 0.0934 | 0.1274 | 4.2482 |
|  | 0.1147 | 0.1835 | 6.1171 |
|  | 0.1005 | 0.1461 | 4.8712 |
| 10M | 0.1586 | 0.2991 | 9.9689 |
|  | 0.1465 | 0.2672 | 8.9072 |
|  | 0.1492 | 0.2743 | 9.1441 |
| 11M | 0.1411 | 0.2530 | 8.4334 |
|  | 0.1292 | 0.2217 | 7.3893 |
|  | 0.124 | 0.2080 | 6.9331 |

Table S3 Data for total triterpenoids content determination.

| Month | OD_550_ | Oleanolic acid (mg/mL) | Total triterpenoids (mg/g) |
| --- | --- | --- | --- |
| 8M | 0.3375 | 0.1050 | 26.2453 |
|  | 0.3583 | 0.1129 | 28.2187 |
|  | 0.3509 | 0.1101 | 27.5166 |
| 9M | 0.3788 | 0.1207 | 30.1637 |
|  | 0.3413 | 0.1064 | 26.6058 |
|  | 0.3729 | 0.1184 | 29.6039 |
| 10M | 0.5831 | 0.1982 | 49.5466 |
|  | 0.6152 | 0.2104 | 52.5921 |
|  | 0.6252 | 0.2142 | 53.5409 |
| 11M | 0.2962 | 0.0893 | 22.3270 |
|  | 0.3096 | 0.0944 | 23.5983 |
|  | 0.325 | 0.1002 | 25.0594 |

Table S4 Data for total polysaccharides content determination.

| Month | OD_490_ | Glucose (mg/mL) | Total polysaccharides (mg/g) |
| --- | --- | --- | --- |
| 8M | 0.4061 | 0.0667 | 5.5583 |
|  | 0.412 | 0.0678 | 5.6533 |
|  | 0.329 | 0.0518 | 4.3163 |
| 9M | 0.7613 | 0.1354 | 11.2799 |
|  | 1.0198 | 0.1853 | 15.4439 |
|  | 1.0812 | 0.1972 | 16.4330 |
| 10M | 1.2577 | 0.2313 | 19.2761 |
|  | 1.2862 | 0.2368 | 19.7352 |
|  | 1.2963 | 0.2388 | 19.8979 |
| 11M | 0.7154 | 0.1265 | 10.5406 |
|  | 0.7064 | 0.1247 | 10.3956 |
|  | 0.6486 | 0.1136 | 9.4645 |

Table S5 Statistics of DAMs by pairwise comparison.

| group name | All sig diff | down regulated | up regulated |
| --- | --- | --- | --- |
| 10M_vs_11M | 323 | 199 | 124 |
| 8M_vs_10M | 286 | 99 | 187 |
| 8M_vs_11M | 216 | 110 | 106 |
| 8M_vs_9M | 363 | 160 | 203 |
| 9M_vs_10M | 295 | 113 | 182 |
| 9M_vs_11M | 373 | 203 | 170 |

Table S6 Correspondence between the number of MetWare database and metabolites.

| Index | Formula | Compounds |
| --- | --- | --- |
| Zmgn000503 | C5H10O4 | 2,3-Dihydroxy-3-Methylbutanoic Acid |
| mws0744 | C15H12O8 | Dihydromyricetin (Ampelopsin) |
| mws1034 | C16H14O5 | Isosakuranetin (5,7-Dihydroxy-4'-methoxyflavanone) |
| Zmdn001863 | C18H14O6 | 2-(7-Dihydroxyl)-benzofuranyl-ferulic acid |
| Hmsn002272 | C15H20O8 | Demethyl coniferin |
| Lmhp002031 | C12H24N2O3 | L-Leucyl-L-Leucine |
| Zmyn000155 | C7H14N2O3 | N-α-Acetyl-L-ornithine |
| Lmhp001670 | C11H22N2O3 | L-Valyl-L-Leucine |
| pmp000172 | C23H24O11 | 5,2'-Dihydroxy-7,8-dimethoxyflavone glycosides |
| Cmsp007228 | C16H14O6 | 7-O-Methyleriodictyol |
| Zmhn001883 | C14H18O9 | Vanillic acid-4-O-glucoside |
| HJN055 | C21H24O10 | Dihydrocharcone-4'-O-glucoside |
| mws2118 | C21H24O10 | Phloretin-2'-O-glucoside (Phlorizin) |
| Lmqp010892 | C35H58O9 | Cyclocarioside J |
| mws0920 | C15H10O7 | Tricetin (5,7,3',4',5'-Pentahydroxyflavone) |
| pmb3107 | C15H20O10 | Glucosyringic Acid |
| Lmtn000940 | C14H18O10 | 1-O-(3,4-Dihydroxy-5-methoxy-benzoyl)-glucoside |
| Lmbp003668 | C20H18O10 | Kaempferol-3-O-arabinoside |
| Lmqp010480 | C35H56O8 | Cyclocarioside Ⅱ |
| Zmhn001926 | C13H16O8 | 1-O-Salicyl-D-glucose |
| pme2165 | C5H4N2O4 | Orotic acid |
| pme1119 | C10H12N4O5 | Inosine |
| Lmsp008392 | C17H14O6 | 4',5-Dihydroxy-3',5'-dimethoxyflavone |
| Zmhp004065 | C18H16O7 | 7,8-Dihydroxy-5,6,4'-trimethoxyflavone |
| Smlp002532 | C20H19O10+ | Cyanidin-3-O-arabinoside |
| Lmqp013019 | C30H48O3 | Epikatonic acid |
| pmn001706 | C30H48O4 | 2-Hydroxyoleanolic acid |
| Zmpn008194 | C30H48O4 | Corosolic acid* |
| pmn001705 | C30H48O4 | 3,24-Dihydroxy-17,21-semiacetal-12(13)oleanolic fruit |
| mws1610 | C30H48O4 | Maslinic acid* |
| Lmzn106284 | C30H48O4 | Alphitolic acid |
| pmn001707 | C30H46O5 | Quillaic acid |
| pmp001079 | C27H30O15 | Luteolin-7-O-neohesperidoside (Lonicerin)* |
| pmp000593 | C27H30O15 | Luteolin-7-O-rutinoside* |
| Lmjp002596 | C26H28O16 | Quercetin-3-O-sambubioside |
| pmp000269 | C30H48O5 | Arjunic Acid |
| Lmsn002887 | C14H16O8 | 1-O-Caffeoyl xylose |
| mws0967 | C20H32O5 | 5,6,15-Trihydroxy-7,9,11,13-eicosatetraenoic acid |
| Lmsn008066 | C30H48O7 | 1β,2α,3α,19α,23-Pentahydroxyurs-12-en-28-oic acid |
| mws0823 | C5H8O3 | 3-Methyl-2-Oxobutanoic acid |
| Lmtn003096 | C26H36O11 | Secoisolariciresinol 4-O-glucoside |
| mws0046 | C27H32O14 | Naringenin-7-O-Neohesperidoside(Naringin)* |
| Lmsn002815 | C27H30O15 | Kaempferol-3-O-rutinoside(Nicotiflorin)* |
| pmb2507 | C5H11O7P | 2-Deoxyribose-1-phosphate |
| pme3146 | C5H10N2O3 | β-Ureidoisobutyric acid |
| mws0145 | C8H8O3 | O-Anisic acid (2-Methoxybenzoic acid) |
| pme3472 | C12H16O7 | Arbutin |
| HJN041 | C21H24O11 | Epicatechin glucoside |
| Lmtp004126 | C24H22O16 | Myricetin-3-O-(6''-malony)glucoside |

Table S7 Statistics of functional annotation of new genes.

| Annotated databases | New Gene Number |
| --- | --- |
| COG_Annotation | 6654 |
| GO_Annotation | 17461 |
| KEGG_Annotation | 14938 |
| KOG_Annotation | 11800 |
| Pfam_Annotation | 16626 |
| Swissprot_Annotation | 15067 |
| TrEMBL_Annotation | 21343 |
| eggNOG_Annotation | 18139 |
| nr_Annotation | 21313 |
| All_Annotated | 21411 |

Table S8 Statistics of functional annotation of miRNA target genes.

| #Anno_Database | Annotated_Number | 300<=length<1000 | length>=1000 |
| --- | --- | --- | --- |
| COG_Annotation | 1460 | 203 | 1257 |
| GO_Annotation | 3177 | 279 | 2895 |
| KEGG_Annotation | 2760 | 257 | 2500 |
| KOG_Annotation | 2112 | 231 | 1879 |
| Pfam_Annotation | 3051 | 254 | 2795 |
| Swissprot_Annotation | 2828 | 252 | 2575 |
| eggNOG_Annotation | 3275 | 273 | 3000 |
| nr_Annotation | 3698 | 306 | 3387 |
| All_Annotated | 3703 | 307 | 3391 |

Table S9 Statistics of DE-miRNAs by pairwise comparison.

| DEG Set | DEG Number | up-regulated | down-regulated |
| --- | --- | --- | --- |
| 10M_vs_11M | 168 | 107 | 61 |
| 8M_vs_10M | 51 | 27 | 24 |
| 8M_vs_11M | 131 | 92 | 39 |
| 8M_vs_9M | 115 | 71 | 44 |
| 9M_vs_10M | 114 | 33 | 81 |
| 9M_vs_11M | 143 | 74 | 69 |
