# Supplementary figures and images for: Metabolome and Whole-Transcriptome Analyses Reveal the Molecular Mechanisms Underlying Hypoglycemic Nutrient Metabolites Biosynthesis in Cyclocarya paliurus Leaves During Different Harvest Stages
Source: Front Nutr. 2022 Feb 28;9:851569. doi: 10.3389/fnut.2022.851569 (PMC8919051; doi:10.3389/fnut.2022.851569)

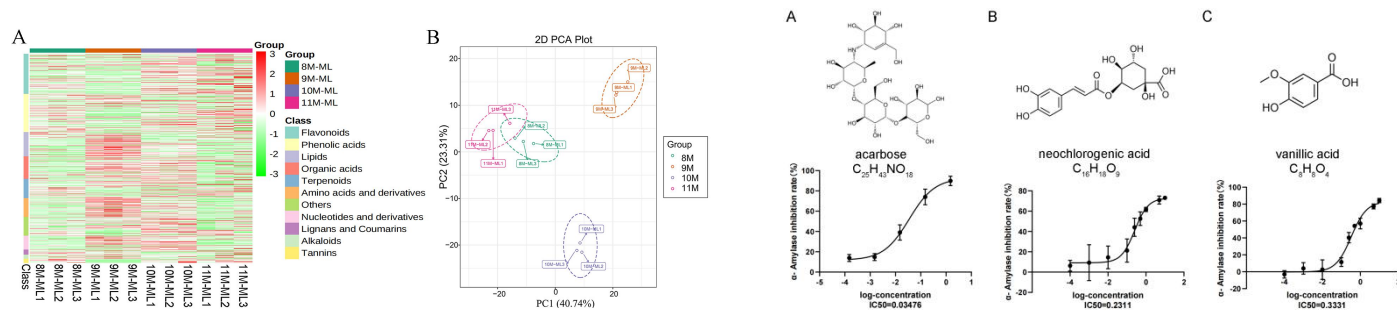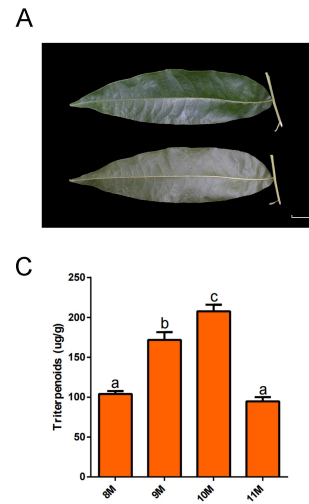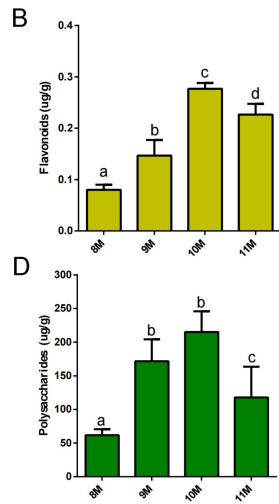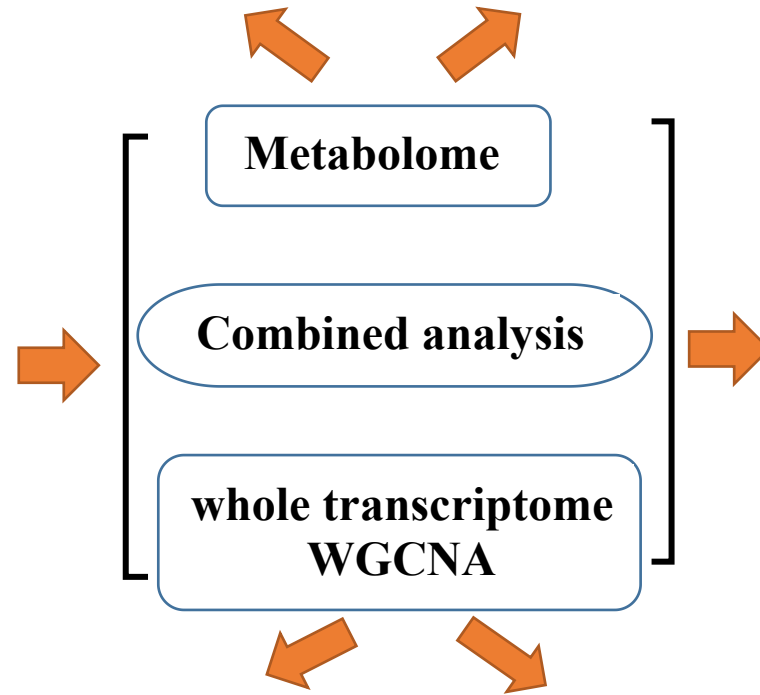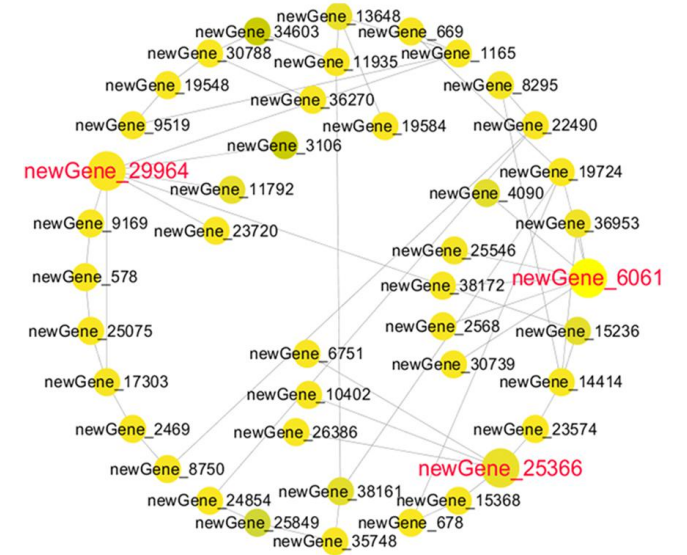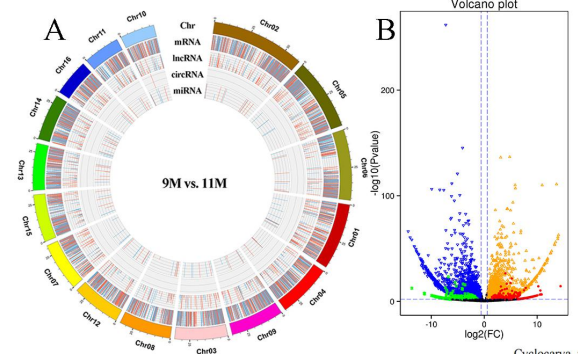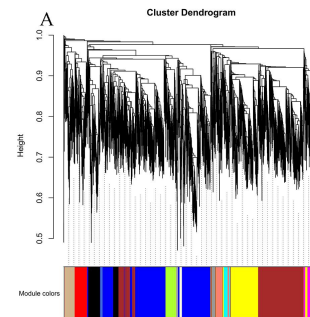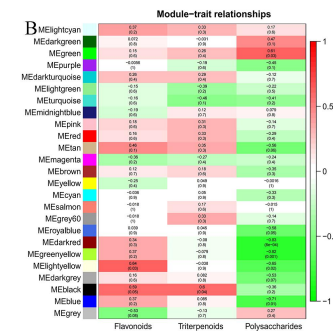

Supplement: Supplementary file 3 [file Image_1.pdf]
